# Concomitant Carboxylate and Oxalate Formation From the Activation of CO_2_ by a Thorium(III) Complex

**DOI:** 10.1002/chem.201604622

**Published:** 2016-10-27

**Authors:** Alasdair Formanuik, Fabrizio Ortu, Christopher J. Inman, Andrew Kerridge, Ludovic Castro, Laurent Maron, David P. Mills

**Affiliations:** ^1^ School of ChemistryThe University of ManchesterManchesterM13 9PLUK; ^2^ Department of Chemistry and BiochemistrySchool of Life SciencesUniversity of SussexBrightonBN1 9QLUK; ^3^ Department of ChemistryLancaster UniversityLancasterLA1 4YBUK; ^4^ LPCNO, CNRA and INSAUniversité Paul Sabatier135 Avenue de RangeuilToulouse31077France

**Keywords:** actinides, reduction, small molecule activation, subvalent compounds, thorium

## Abstract

Improving our comprehension of diverse CO_2_ activation pathways is of vital importance for the widespread future utilization of this abundant greenhouse gas. CO_2_ activation by uranium(III) complexes is now relatively well understood, with oxo/carbonate formation predominating as CO_2_ is readily reduced to CO, but isolated thorium(III) CO_2_ activation is unprecedented. We show that the thorium(III) complex, [Th(Cp′′)_3_] (**1**, Cp′′={C_5_H_3_(SiMe_3_)_2_‐1,3}), reacts with CO_2_ to give the mixed oxalate‐carboxylate thorium(IV) complex [{Th(Cp′′)_2_[κ^2^‐O_2_C{C_5_H_3_‐3,3′‐(SiMe_3_)_2_}]}_2_(μ‐κ^2^:κ^2^‐C_2_O_4_)] (**3**). The concomitant formation of oxalate and carboxylate is unique for CO_2_ activation, as in previous examples either reduction or insertion is favored to yield a single product. Therefore, thorium(III) CO_2_ activation can differ from better understood uranium(III) chemistry.

There has been an international drive to reduce emissions of CO_2_ through cleaner energy generation since its identification as a key contributor to global warming.^1^ In tandem the employment of CO_2_ as a C_1_ feedstock for fine chemical (by direct insertion into organic molecules)^2^ and liquid fuel (via reduction to CO for Fischer–Tropsch processes)^3^ synthesis have rapidly expanded to complement the optimized photosynthetic pathways employed by nature.^4^ Early d‐transition metal complexes have received most attention for CO_2_ activation as their inherent oxophilicity is advantageous in overcoming the considerable thermodynamic and kinetic barriers in this process.^5^ Similarly, actinides are highly oxophilic, so CO_2_ activation by U^III^ complexes is also developing rapidly^6^ and proof of concept catalytic processes have been disclosed.^7^ The mapping of U^III^‐mediated CO_2_ activation by DFT calculations has provided key insights into possible mechanistic pathways.^8^ In contrast, Cloke reported the only example of CO_2_ activation by a putative Th^III^ intermediate^9^ as Th^III^ small molecule activation is in its infancy.^10^, ^11^ Herein we report the first reaction of an isolated Th^III^ complex with CO_2_, and CS_2_ for comparative studies.

[Th(Cp′′)_3_] (**1**, Cp′′={C_5_H_3_(SiMe_3_)_2_‐1,3}) reacts with 0.5 to 10 equivalents of CS_2_ to give [{Th(Cp′′_3_)}_2_(μ‐κ^1^:κ^2^‐CS_2_)] (**2**) as the only isolable product in 45 % yield (Scheme 1; see the Supporting Information for full details). This reaction is consistent with U^III^ chemistry as the double reduction of CS_2_ by [U(Cp′)_3_] (Cp′=C_5_H_4_SiMe_3_) yields [{U(Cp′_3_)}_2_(μ‐κ^1^:κ^2^‐CS_2_)].^12^ However, **1** reacts with excess CO_2_ to give [{Th(Cp′′)_2_[η^2^‐O_2_C{C_5_H_3_‐3,3′‐(SiMe_3_)_2_}]}_2_(μ‐κ^2^:κ^2^‐C_2_O_4_)] (**3**) in 65 % yield (Scheme 1), in contrast to the U^III^ reduction of CO_2_ by [U(Cp′)_3_] to afford [{U(Cp′)_3_}_2_(μ‐O)] and CO.^13^ The FTIR spectrum of **3** has absorptions at 1653 cm^−1^ and 1560 cm^−1^ that can be attributed to asymmetric C−O stretches of the oxalate and carboxylate groups respectively.^14^ The reaction of **2** with CO_2_ gave a mixture of products including carboxylate (see the Supporting Information).

**Scheme 1:**
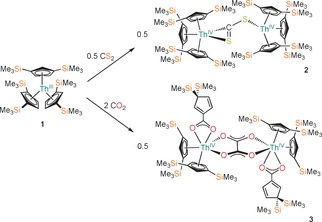
Synthesis of **2** and **3** from **1**.

The solid state structures of **2** and **3⋅**2C_7_H_8_ were determined by single crystal XRD (Figure 1. A polymorph **2 b⋅**2C_6_H_14_ was also obtained; see the Supporting Information). The (μ‐CS_2_)^2−^ unit in **2** was disordered over two positions so only the major component is discussed here. This fragment binds in an asymmetrical μ‐κ^1^:κ^2^‐fashion [S−C: 1.644(11) and 1.717(10) Å; S‐C‐S: 124.4(7)°], in common with the motif seen for [{U(Cp′_3_)}_2_(μ‐κ^1^:κ^2^‐CS_2_)] [S−C: 1.464(19) and 1.831(19) Å; S‐C‐S: 131.7(13)°]^12^ and similar to that seen for [{U[OSi(O*t*Bu)_3_]_3_}_2_(μ‐κ^2^:κ^2^‐CS_2_)] [S−C: 1.594(12) and 1.748(11) Å; S‐C‐S: 131.6(8)°].^15^ The oxalate of **3** has similar metrical parameters to those seen in [{Th(COT^TIPS2^)(Cp*)}_2_(μ‐κ^2^:κ^2^‐C_2_O_4_)].^9^ The carboxylate ligand exhibits both C−C and C=C lengths in the C_5_ ring and a geminal 3,3′‐disilane. The C−O_carboxylate_ [1.284(5) and 1.263(5) Å] lengths evidence delocalization about the carboxylate framework, although the binding is asymmetric due to sterics [Th−O_carboxylate_ 2.400(3) and 2.484(2) Å]. The electronic structures of **2** and **3** were characterized at the DFT level, employing the B3LYP exchange‐correlation functional and a polarized split‐valence basis set for structural optimizations. Structural parameters of **3** were in good agreement with experiment (see the Supporting Information for full details).

**Figure 1:**
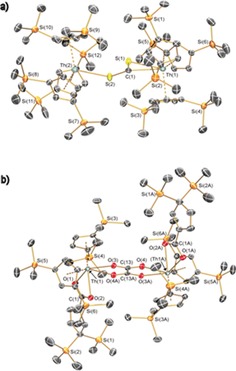
Molecular structure of a) **2** and b) **3**.2C_7_H_8_ with selected atom labelling and displacement ellipsoids set to 30 % probability level. Hydrogen atoms, minor disorder components and lattice solvent omitted for clarity.

We postulated that **3** forms via a [{Th(Cp′′)_3_}_2_(μ‐CO_2_)] intermediate that is analogous to **2**. The bulky Cp′’ ligands hinder the elimination of CO and the formation of [{Th(Cp′′)_3_}_2_(μ‐O)],^16^ so a second molecule of CO_2_ reacts with the (μ‐CO_2_)^2−^ fragment to give an oxalate. There are many examples of sterically demanding ligands promoting oxalate formation over a μ‐oxo or carbonate in f‐block CO_2_ activation.^8^, ^17^ Subsequent insertion of CO_2_ into a Th–Cp′’ moiety and silyl/proton migration yields **3**. The insertion of CO_2_ into lanthanide‐Cp^R^ bonds to form carboxylates has been postulated not to require steric strain to proceed.^18^ Additional experiments were performed to probe the mechanism of formation of **3** (see the Supporting Information for full details). A Toepler pump was used to react **1** with 1 or 2 equivalents of CO_2_ or ^13^CO_2_ at −78 °C, and **3**/**3‐^13^C** was the only identifiable product by ^1^H and ^13^C{^1^H} NMR spectroscopy in all cases. The reaction of **1** with supercritical CO_2_ was monitored by ^1^H NMR spectroscopy, and comparison with an authentic sample showed the formation of **3**. Minor products in all reaction mixtures could not be identified. In situ FTIR spectroscopy was used to monitor the conversion of **1** to **3** at −78 °C in methylcyclohexane with stoichiometric CO_2_. No intermediates could be detected but the CO_2(g)_ absorption at 2338 cm^−1^ diminished on slow warming to room temperature, coincident with the ingress of an oxalate absorption at 1653 cm^−1^ that is seen in the FTIR spectrum of crystalline **3**. The experiment was repeated with ^13^CO_2_ and the oxalate absorbance of **3‐^13^C** was observed at 1609 cm^−1^, consistent with reduced mass considerations.

Given that no intermediates could be detected experimentally, we performed DFT studies to rationalize this unusual mechanism. Figure 2 shows the calculated enthalpy reaction profile for the formation of **3**, with the double reduction of CO_2_ to give a μ‐κ^1^:κ^2^‐CO_2_ dinuclear Th^IV^ complex the proposed first step based on the analogous CS_2_ reaction as well as CO_2_ reactivity reported with other actinide complexes.^8^, ^12^, ^15^, ^17^ The oxalate formation invokes nucleophilic attack of a CO_2_ molecule by a dimetalloxycarbene intermediate [{Th(Cp′′_3_)}_2_(μ‐κ^1^:κ^1^‐CO_2_)] (C2) in a carbenic fashion, which has previously only been seen in d‐block CO_2_ activation for Ti^IV^.^19^ No pre‐interaction is required between the Th^IV^ centers and the second CO_2_ molecule, which is in contrast with all previous examples of Sm^II[17a–c]^ and U^III[17d]^ oxalate formation. The resultant *cis*‐μ‐κ^1^:κ^1^‐C_2_O_4_ transition state (TS1) is one of several possible conformers that have Δ_r_ *H*° values within the estimated error of the calculation (ca. 3 kcal mol^−1^) of each other, thus we do not comment on this further. Rearrangement of the oxalate to a *trans*‐μ‐κ^1^:κ^1^‐binding mode increases the steric demands about the Th^IV^ centers (C3). The potential energy surface for these rearrangements is very flat and despite our efforts it was not possible to locate a transition state. This leads to insertion of CO_2_ at a single position of a Th–Cp′’ moiety at each Th^IV^ center (TS2) as the silicon centers stabilize negative charge at the beta position, allowing the best overlap with the empty orbital of CO_2_. These insertions are accompanied by the rearrangement of the oxalate to a μ‐κ^2^:κ^2^‐binding mode. Subsequent proton and silyl group migrations in the dearomatized Cp′′ rings give the observed product **3** at an energetic minimum.

**Figure 2:**
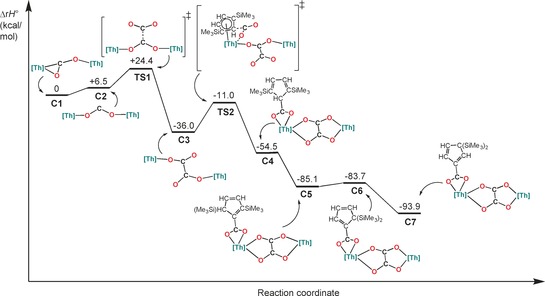
Computed enthalpy reaction profile for the formation of **3**.

To conclude we have shown that although CS_2_ activation by [Th(Cp′′)_3_] is analogous to that seen for a similar U^III^ system, the mechanism by which it reacts with CO_2_ to form a mixed oxalate/carboxylate product has no precedent in U^III^ chemistry, in which CO_2_ reduction (and subsequent carbonate formation depending on the supporting ligands) predominates. We probed this reaction to show that the oxalate is generated by a mechanism only seen previously in d‐block chemistry, whereas the carboxylate forms via a route only seen before in f‐element chemistry for lanthanide complexes. This shows that Th^III^ small molecule activation can furnish results that complement and contrast with uranium, lanthanide and d‐transition metals. Future studies will target heteroallene activation by Th^III^ complexes supported by different ligand systems to test the generality or divergence of these processes.

## Experimental Section

Full synthetic details, characterization data and computational data for **2** and **3** is available in the Supporting Information. Additional research data supporting this publication are available from The University of Manchester eScholar repository at DOI: 10.15127/1.302780.

## Supporting information

As a service to our authors and readers, this journal provides supporting information supplied by the authors. Such materials are peer reviewed and may be re‐organized for online delivery, but are not copy‐edited or typeset. Technical support issues arising from supporting information (other than missing files) should be addressed to the authors.

Supplementary
