# Supplementary material for: Concomitant Carboxylate and Oxalate Formation From the Activation of CO2 by a Thorium(III) Complex
Source: Chemistry. 2016 Oct 27;22(50):17976–9. doi: 10.1002/chem.201604622 (PMC5215673; doi:10.1002/chem.201604622)
Supplement: Supplementary file 1 — Supplementary [file CHEM-22-17976-s001.pdf]

CHEMISTRY
A European Journal

Supporting Information

Concomitant Carboxylate and Oxalate Formation From the Activation of CO_2 by a Thorium(III) Complex

Alasdair Formanuik,[a] Fabrizio Ortu,[a] Christopher J. Inman,[b] Andrew Kerridge,[c] Ludovic Castro,[d] Laurent Maron,*[d] and David P. Mills*[a]

chem_201604622_sm_miscellaneous_information.pdf

Contents

1. **Experimental procedures** **S2**
2. **IR spectroscopy** **S5**
3. **NMR spectroscopy** **S8**
4. **Crystallographic details** **S15**
5. **Computational details** **S17**
6. **References** **S61**

1. Experimental Procedures

Caution – thorium-232 is a weak α-emitter ($t_{1/2}$ 1.41 x 10^{10} years), therefore all manipulations should be performed in suitable laboratories that have been designated for radiochemical use, and α-counting equipment should be available. All manipulations were performed using standard Schlenk techniques or in an Inert Purelab HE 2GB glove box. Toluene, methylcyclohexane (MeCy), [D_6]benzene and [D_8]toluene were dried by refluxing over potassium, stored over potassium mirrors and degassed before use. CS_2 was dried over CaH_2 and vacuum distilled, CO_2 was used as supplied by BOC. [Th(Cp")$_3$] (**1**)[1] was prepared according to published procedures. ^{1}H, ^{13}C{^{1}H} and ^{29}Si{^{1}H} NMR spectra were recorded on a Bruker DPX400 spectrometer operating at 400.2, 100.6 and 79.5 MHz, respectively; chemical shifts are quoted in ppm and are relative to TMS. FTIR spectra were either recorded as Nujol mulls in KBr discs on a Shimadzu IRAffinity-1S spectrometer, or on a Mettler-Toledo Autochem ReactIR 15. Elemental microanalysis was carried out by Mrs Anne Davies and Mr Martin Jennings at the Microanalysis Service, School of Chemistry, the University of Manchester, UK. Low carbon values were obtained upon repeating elemental analysis experiments multiple times on different batches of **2** and **3** and is ascribed to them being silicon-rich molecules, as previously reported.[23]

Stoichiometric gas transfer was *via* a Toepler pump with calibrated (by transfer of xenon into a receiving vessel of accurately known volume, and differential weighing) delivery pressure. Control of stoichiometry of the gas reactions was achieved by the use of standard 5 mm (for the NMR and IR studies) or shortened 10 mm (for preparative scale) Young's NMR tubes of accurately known volume containing measured volumes (microliter syringe) of solvent (and therefore of known headspace). Reactions were typically performed using 10 mg of **1**, with the increased impurities observed in these NMR spectra compared with bulk scales attributed to decomposition products from trace O_2/H_2O making up a larger proportion of the reaction mixtures.

Reactions with supercritical CO_2 (99.999% BOC) were performed in a 10 mm sapphire NMR tube equipped with a titanium valve, connected to a high pressure stainless steel line equipped with a high pressure piston pump (High Pressure Equipment Company). 30 mg of the compound was loaded in the sapphire tube in the glove box. This was then attached to the high

pressure line and evacuated for approximately 30 minutes. It was then chilled to about 5 ºC (water bath with ice) and CO_2 was administered in the vessel by means of the HP pump until liquid CO_2 started condensing (50-55 bar). The reaction vessel was then isolated from the CO_2 supply and was heated at 40 ºC by means of a water bath for at least 2 hours. Heating was ceased and the vessel allowed to cool to room temperature. Excess CO_2 was vented carefully and the reaction vessel was evacuated before being transferred to the glove box where it was extracted in the appropriate solvent and analyzed.

[{Th(Cp″)$_3$}$_2$(μ-κ^1:κ^2-CS_2)] (**2**). A solution of CS_2 (38 mg, 30 μl, 0.5 mmol) in toluene (10 ml) was added to a –78 °C solution of **1** (0.404 g, 0.47 mmol) in toluene (20 ml), with an immediate color change from blue to dark yellow. The reaction mixture was allowed to warm to room temperature and stirred for 18 hours. The solution was filtered, and volatiles removed *in vacuo*. The yellow residue was dissolved in 1 ml of pentane and stored at 4 °C for 16 hours to yield **2** as yellow-brown crystals (0.217 g, 0.22 mmol, 51 %). ^{1}H NMR (400.2 MHz, [D$_6$]benzene, 25°C): δ = 0.50 (s, 54 H; SiMe$_3$), 0.60 (s, 54 H; SiMe$_3$), 7.16 (br m, 12 H; Cp-*H*), 7.34 (s, 6 H; Cp-*H*); ^{13}C{^{1}H} NMR (100.6 MHz, [D$_6$]benzene, 25°C): δ = 2.72 (SiMe$_3$), 2.98 (SiMe$_3$), 131.44 (Cp-*C*), 135.16 (Cp-*C*H), 136.57 (Cp-*C*), 140.00 (Cp-*C*H), *C*S$_2$ not observed; ^{29}Si{^{1}H} NMR (79.5 MHz, [D$_6$]benzene, 25°C): δ = –7.72, –9.08 (*Si*Me$_3$); IR (Nujol): *v*bar = 1248 (s), 1078 (s), 961 (m), 920 (m), 837 (m), 752 (m), 640 (w) cm^{-1}; elemental analysis calcd (%) for $C_{73}H_{134}N_2Si_{12}Th_2$: C 44.78, H 7.07; found: C 43.34, H 7.07.

[{Th(Cp″)$_2$[η^2-O_2C{C_5H_3-3,3′-(SiMe$_3$)$_2$}]}$_2$(μ-κ^2:κ^2-C_2O_4)] (**3**). A toluene solution of **1** (0.430 g, 0.5 mmol) was thoroughly degassed and cooled to –78 °C. The solution was exposed to CO_2 (1 bar) and slowly allowed to warm to 0 °C, forming a pale yellow reaction mixture. The solution was stirred at 0 °C for a further 30 minutes before the volatiles were removed *in vacuo* and the white solid recrystallized from toluene (1 ml) to yield colorless crystals of **3** (0.325 g, 0.32 mmol, 65 %). ^{1}H NMR (400.2 MHz, [D$_6$]benzene, 25°C): δ = 0.02 (s, 36 H; (SiMe$_3$)$_2$), 0.50 (s, 36 H; SiMe$_3$), 0.51 (s, 36 H; SiMe$_3$), 6.39 (m, 2 H; Cp-*H*), 6.88 (m, 4 H; Cp-*H*), 7.14 (m, 8 H; Cp-*H*), 7.33 (m, 2 H; Cp-*H*), 7.71 (m, 2 H; Cp-*H*); ^{13}C{^{1}H} NMR (100.6 MHz,

[D_6]benzene, 25°C): δ = –0.20 ($(SiMe_3)_2$), 1.19 ($SiMe_3$), 1.39 ($SiMe_3$), 62.15 ($C(SiMe_3)_2$), 126.39 (Cp-*C*H), 127.45 (Cp-*C*H), 130.27 (H*C*{$C_5H_3(SiMe_3)_2$-3,3}), 137.14 (Cp-*C*H), 137.81 (H*C*{$C_5H_3(SiMe_3)_2$-3,3}), 140.02 (Cp-*C*), 148.46 (H*C*{$C_5H_3(SiMe_3)_2$-3,3}), 172.06 (C_2O_4), 178.16 (O_2C-$C_5H_3(SiMe_3)_2$-3,3). (O_2C-*C*-$C_5H_3(SiMe_3)_2$-3,3) not observed; $^{29}Si\{^1H\}$ NMR (79.5 MHz, [D_6]benzene, 25°C): δ = –0.02 ($(SiMe_3)_2$), –8.82 ($SiMe_3$), –9.25 ($SiMe_3$); IR (Nujol): *v*bar = 1653 (s, $C\text{-}O_{oxalate}$), 1560 (s, $C\text{-}O_{carboxylate}$), 1365 (w), 1254 (s), 1171 (s), 1082 (s), 974 (s), 922 (s), 835 (m), 756 (s) cm^{-1}; elemental analysis calcd (%) for $C_{70}H_{126}O_8Si_{12}Th_2$: C 44.32, H 6.70; found: C 43.34, H 6.72.

Reaction of 2 with CO_2. A toluene solution of **2** (0.750 g, 0.4 mmol) was thoroughly degassed and cooled to –78 °C. The solution was exposed to CO_2 (1 bar) and slowly allowed to warm to room temperature, forming a dark red reaction mixture. The volatiles were removed *in vacuo* yielding a red solid. No crystalline products could be obtained but ^1H and ^{13}C NMR and FTIR spectra of the crude mixture was obtained. In the ^{13}C NMR spectrum diagnostic peaks indicating formation of the (O_2C-$C_5H_3(SiMe_3)_2$-3,3) fragment were observed, such as the quaternary O_2C signal at 178.08 ppm. The absorbance at 1562 cm^{-1} in the FTIR spectrum can be attributed to the asymmetric C–O stretch of a carboxylate group as in **3**. No signals consistent with oxalate formation were observed in either the ^{13}C NMR or FTIR spectra. As the CS_2 signal could not be observed in the ^{13}C NMR spectrum, the persistence of the (μ-κ^1:κ^2-CS_2) motif cannot be unequivocally confirmed.

2. IR Spectroscopy

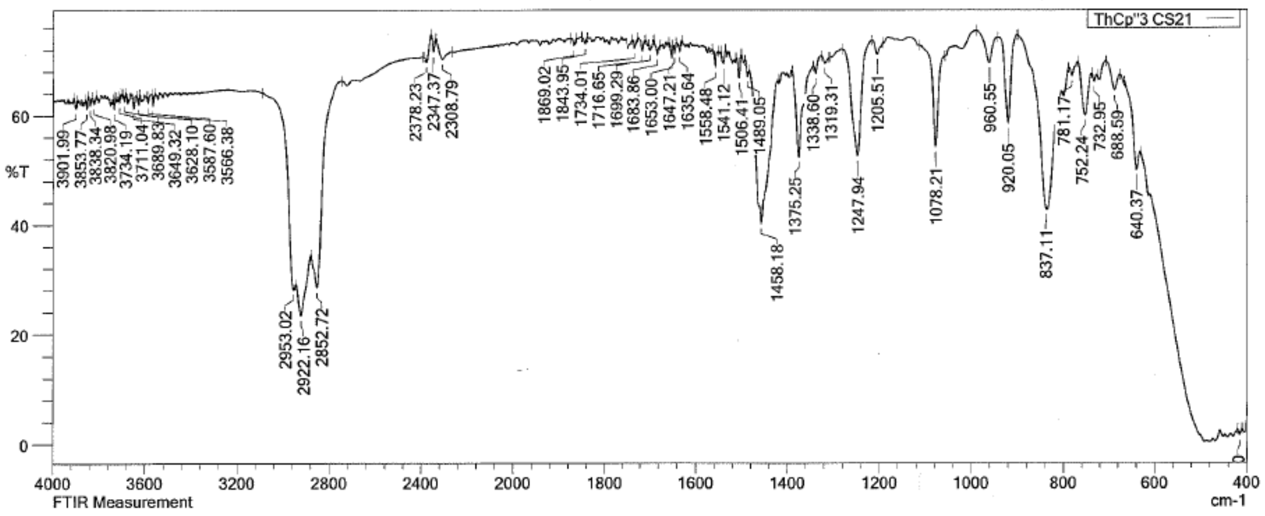

Figure S1. The FTIR spectrum of **2** as a nujol mull.

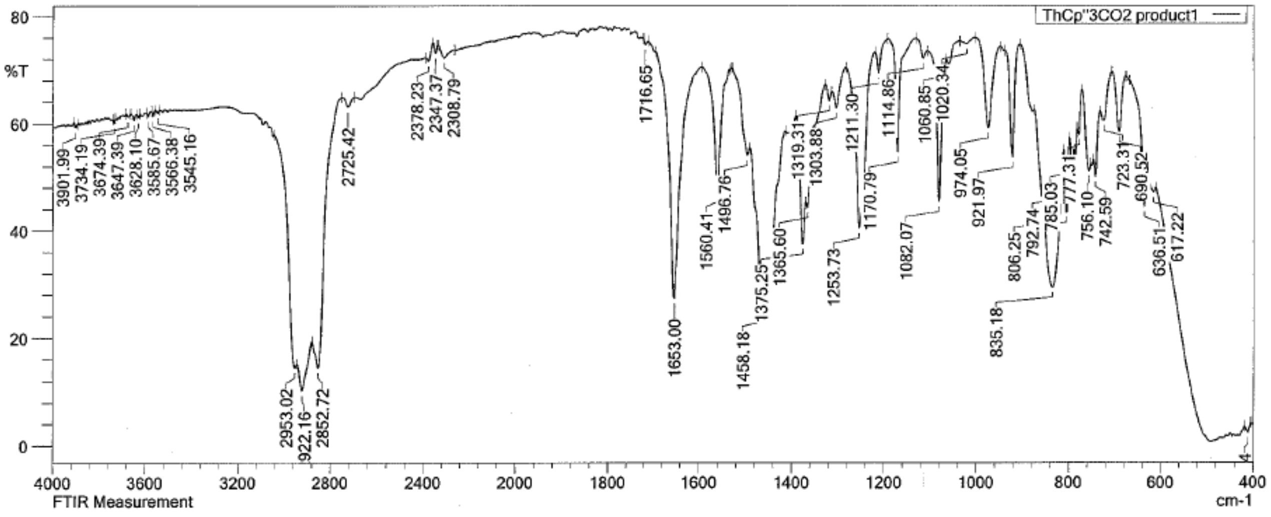

Figure S2. The FTIR spectrum of **3** as a nujol mull.

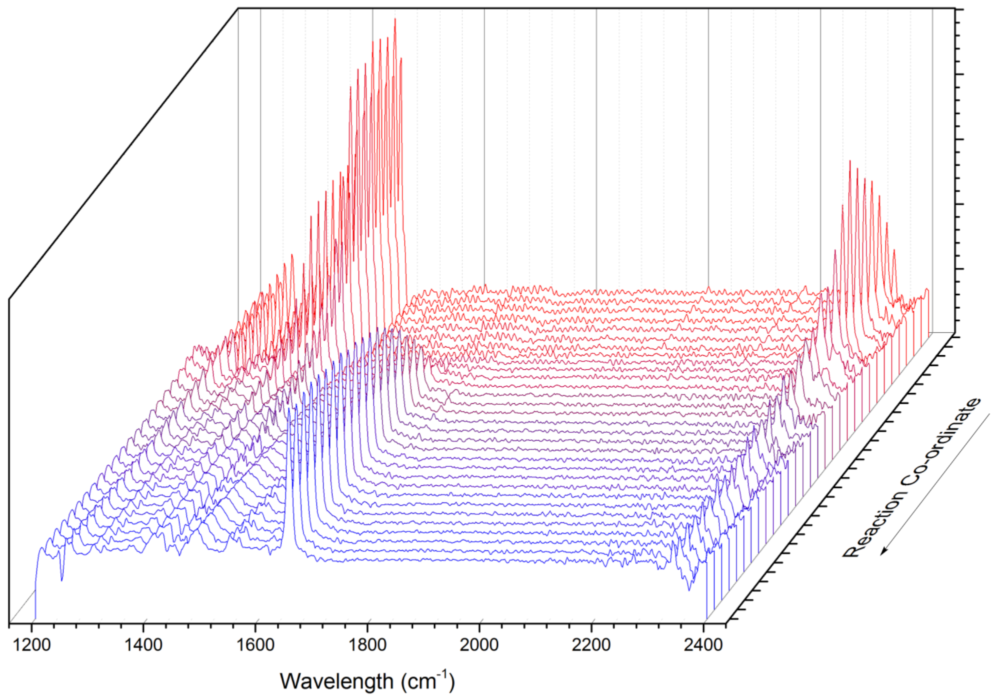

Figure S3. The TRIR spectra of the reaction of **1** and $^{12}CO_2$ (1:2) (in MeCy). The reaction co-ordinate is from back to front (Red to Blue). The peaks at 1445 cm^{-1} and 2338 cm^{-1} which are observed to diminish are from free CO_2. The ingress of the peak at 1653 cm^{-1} is attributed to the formation of **3**.

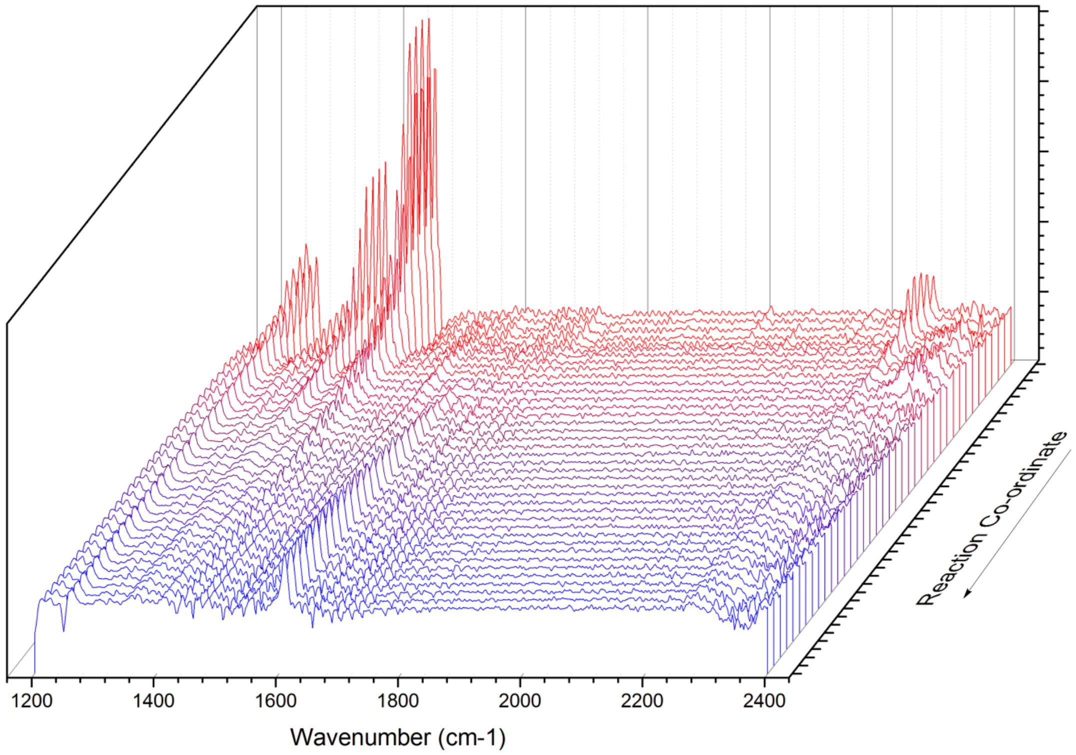

Figure S4. The TRIR spectra of the reaction of **1** and $^{13}CO_2$ (1:2) (in MeCy). The reaction co-ordinate is from back to front (Red to Blue). The peaks at 1446 cm^{-1} and 2273 cm^{-1} which are observed to diminish are from free $^{13}CO_2$. The ingress of the peak at 1609 cm^{-1} is attributed to the formation of **3-^{13}C**.

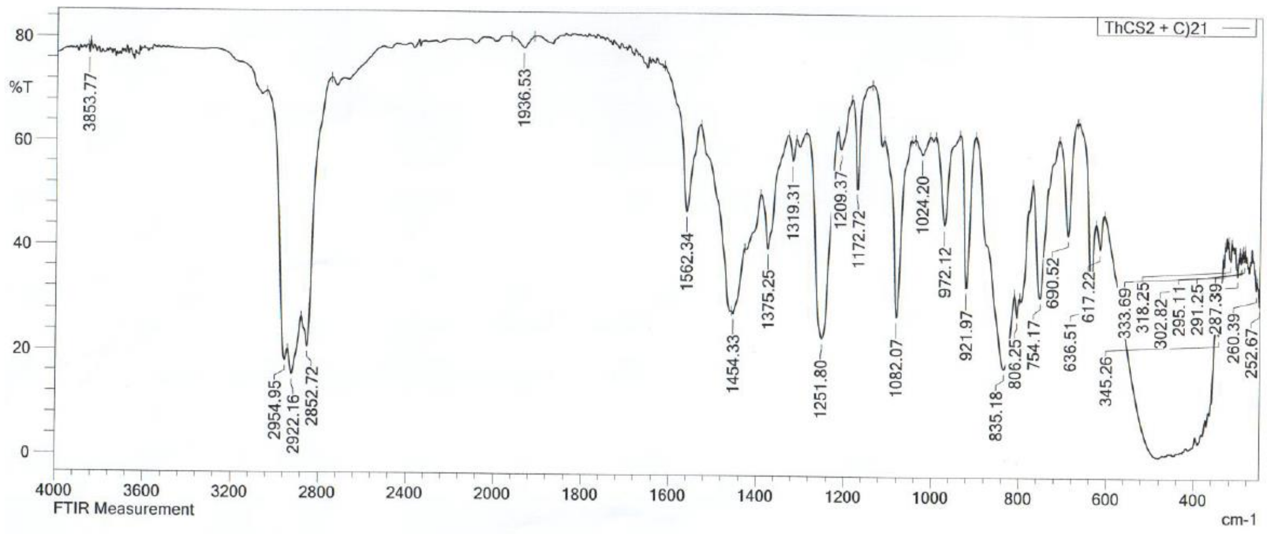

Figure S5. The FTIR spectrum of the reaction mixture of **2** and CO_2 as a nujol mull.

3. NMR spectroscopy

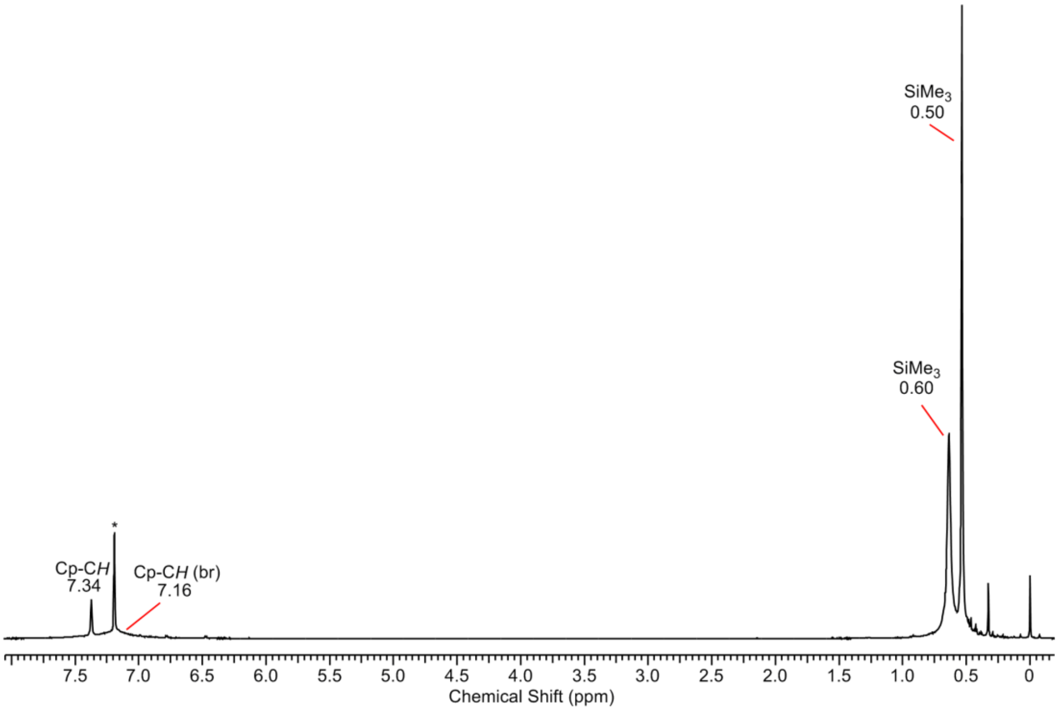

Figure S6. ^{1}H NMR spectrum of **2** at 298 K in [D_6]benzene (*).

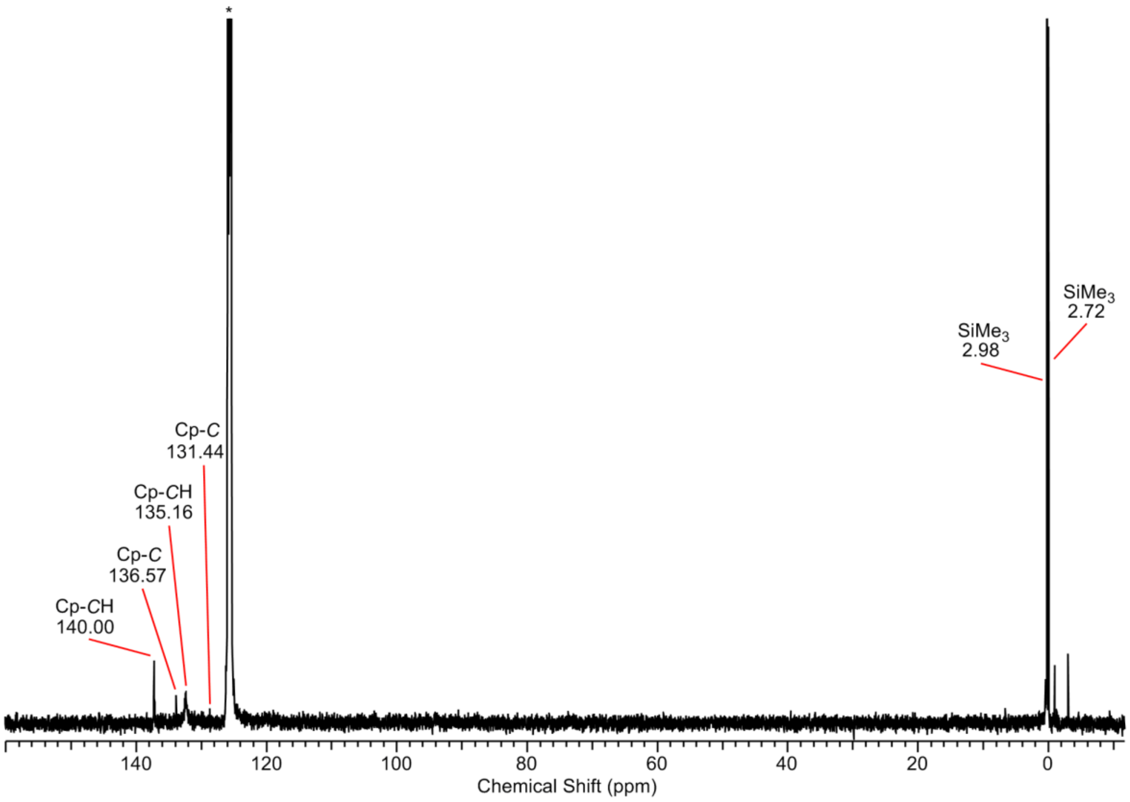

Figure S7. ^{13}C NMR spectrum of **2** at 298 K in [D_6]benzene (*).

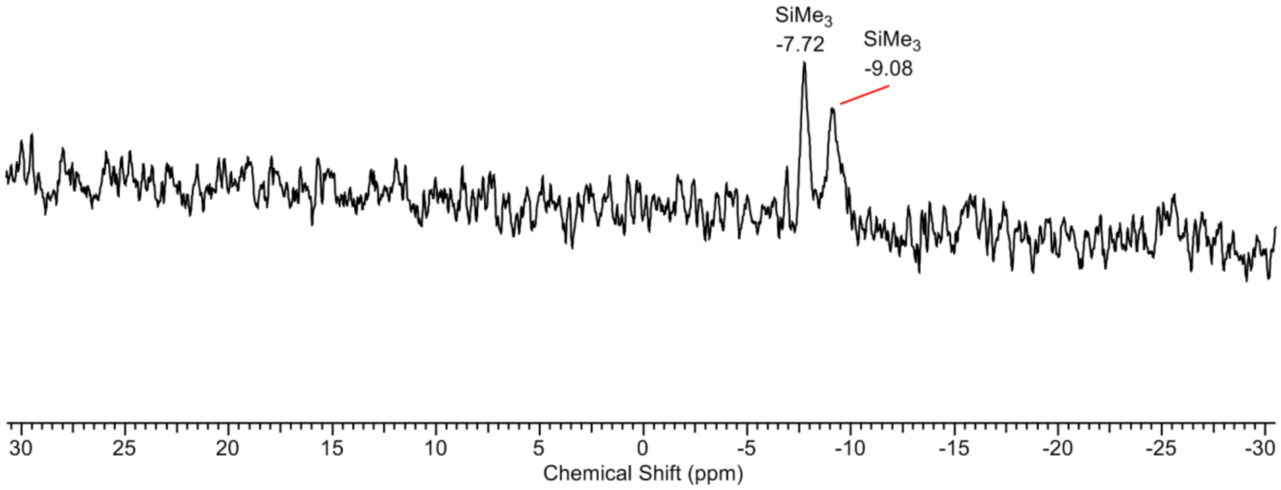

Figure S8. ^{29}Si NMR spectrum of **2** at 298 K in [D$_6$]benzene.

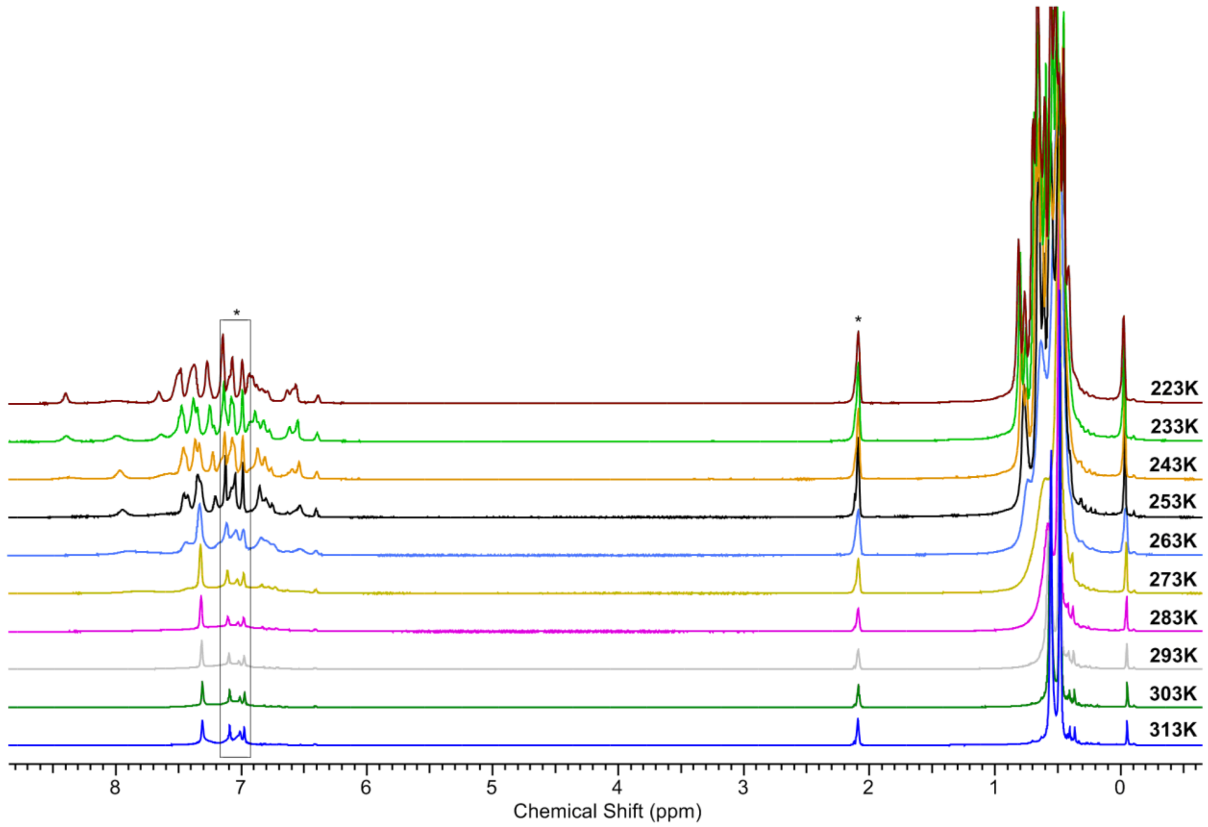

Figure S9. VT ^{1}H NMR spectra of **2** from 223K-313 K in [D$_8$]toluene (*).

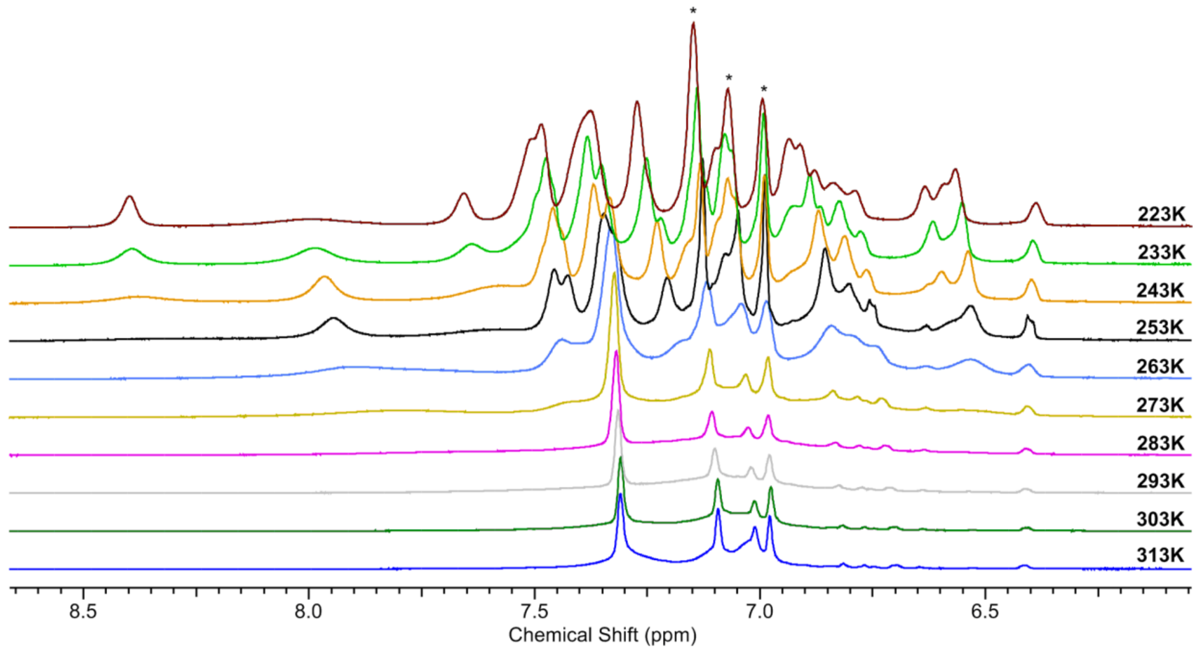

Figure S10. VT ^{1}H NMR spectra of the aromatic region of **2** from 223K-313 K in [D_8]toluene (*).

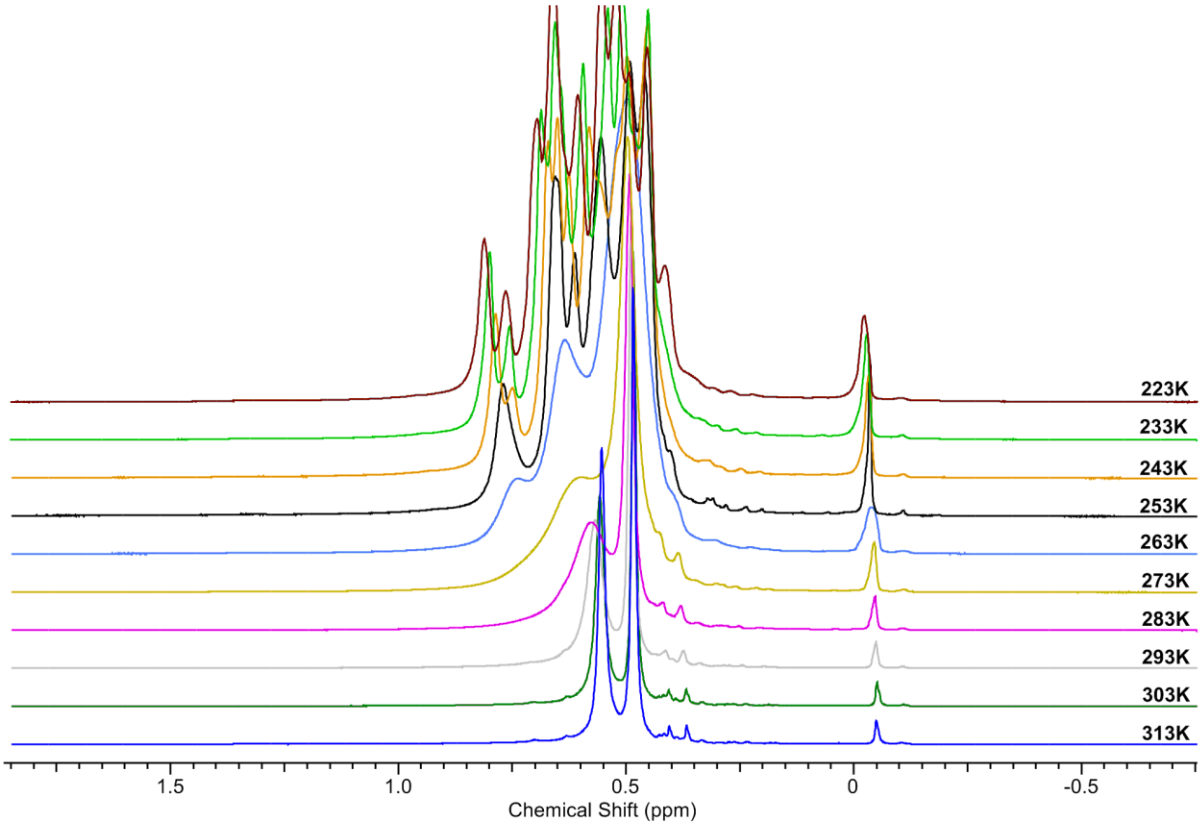

Figure S11. VT ^{1}H NMR spectra of the silyl region of **2** from 223K-313 K in [D_8]toluene.

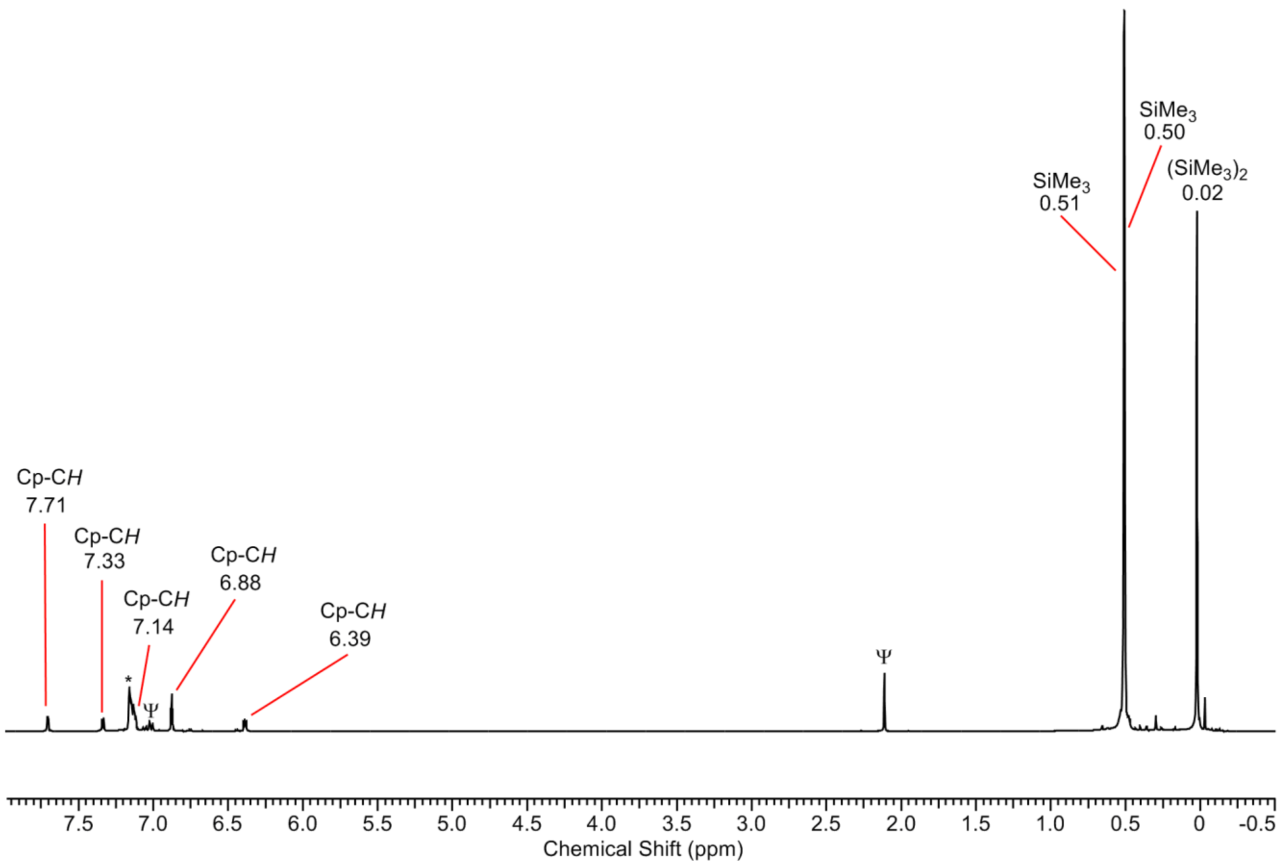

Figure S12. ^{1}H NMR spectrum of **3** at 298 K in [D_6]benzene (*). Ψ denotes residual toluene.

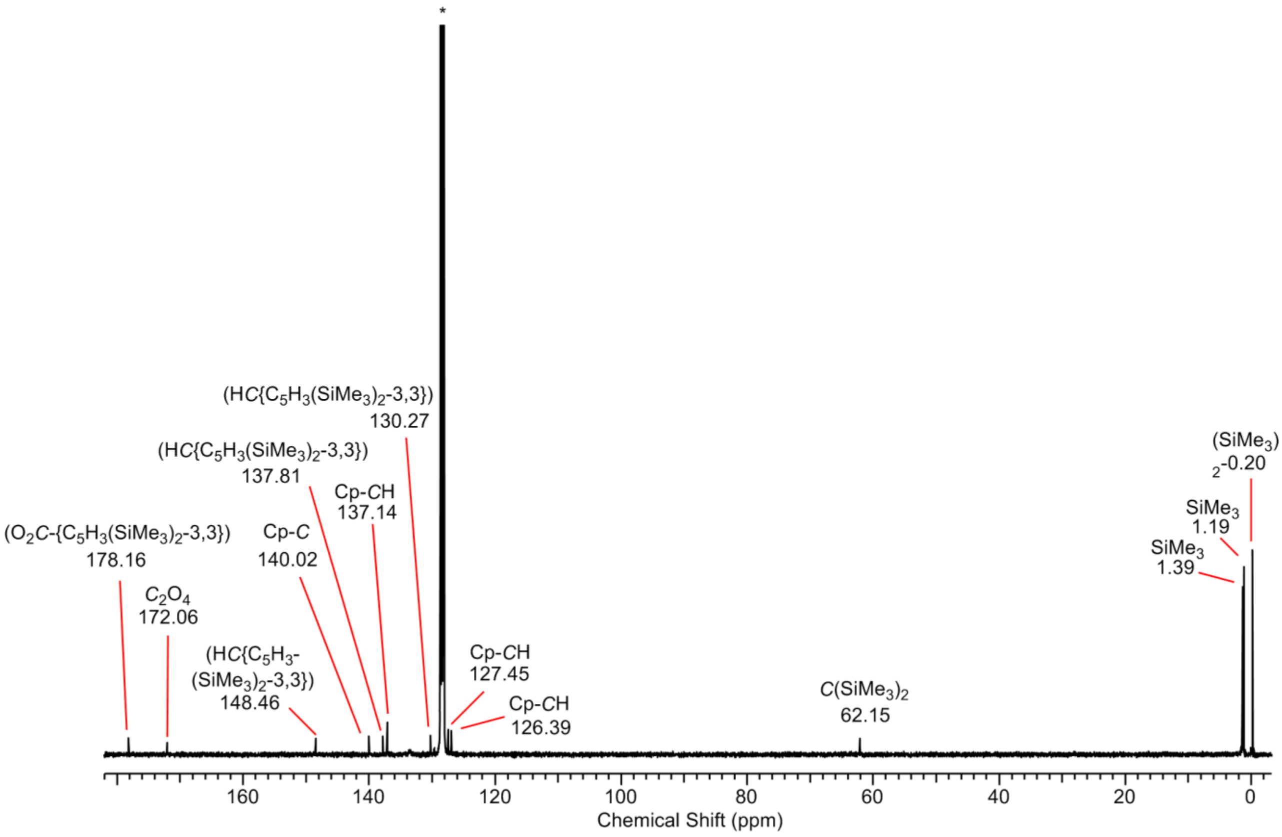

Figure S13. ^{13}C NMR spectrum of **3** at 298 K in [D_6]benzene (*).

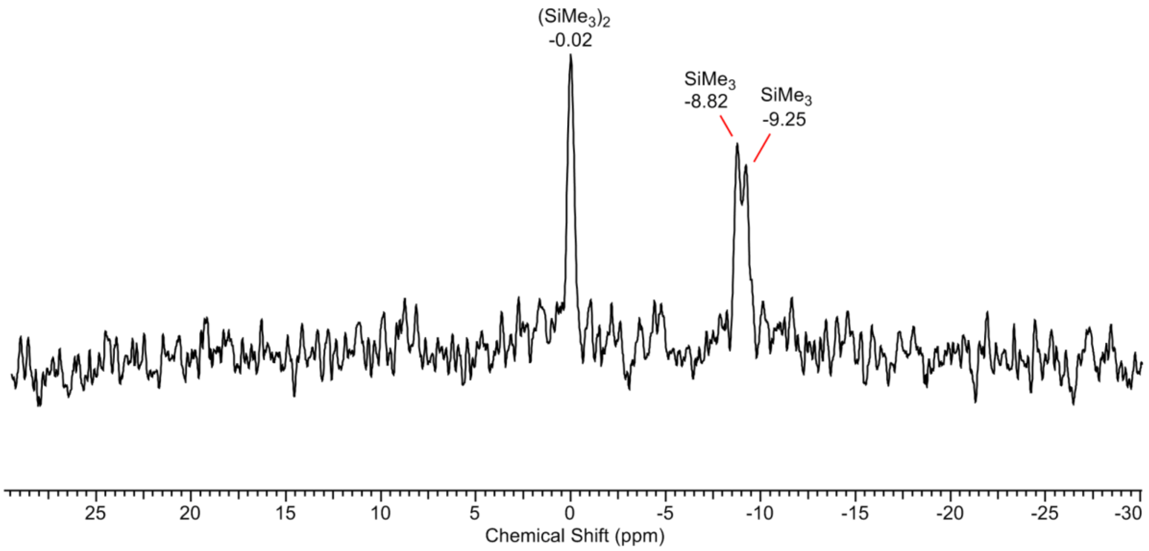

Figure S14. ^{29}Si NMR spectrum of **3** at 298 K in [D$_6$]benzene.

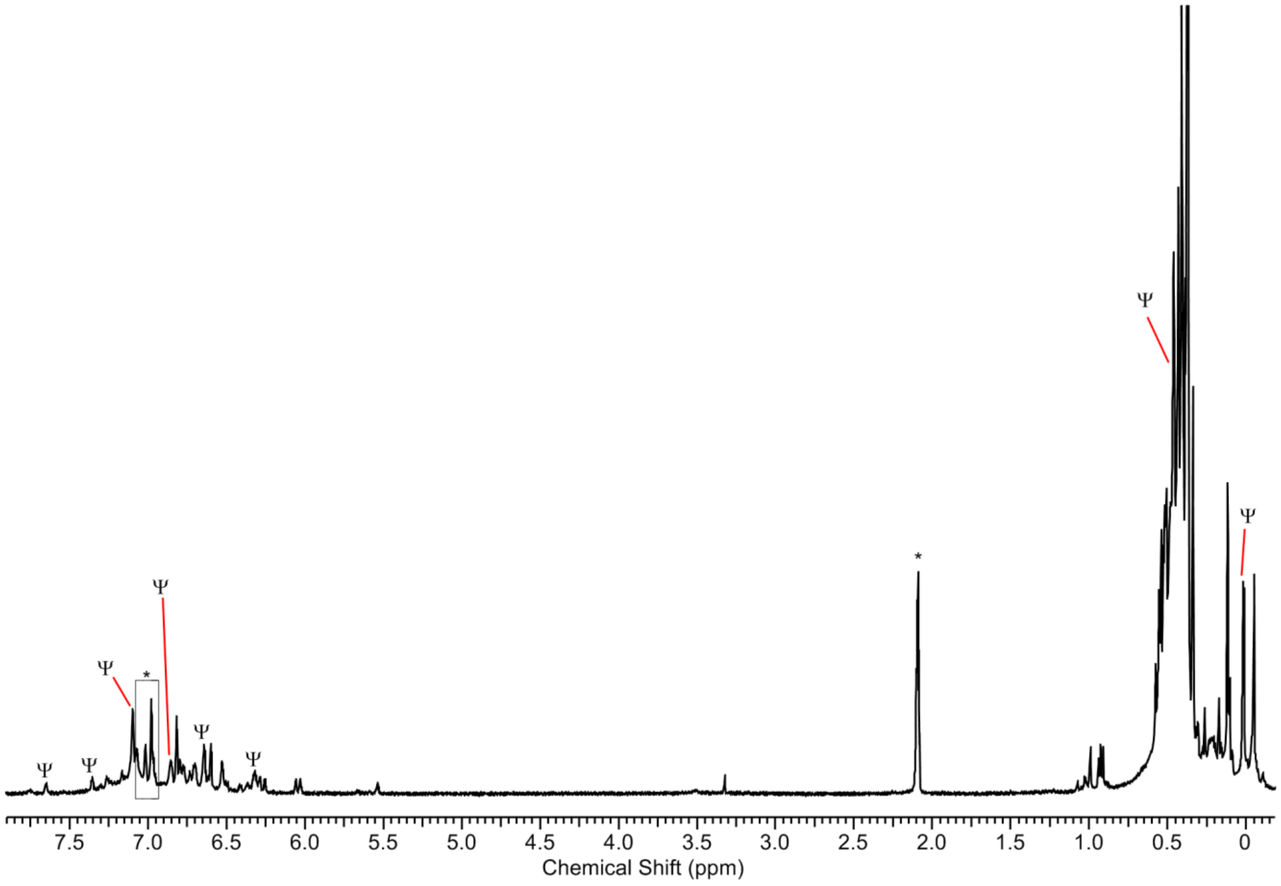

Figure S15. ^{1}H NMR spectrum of the reaction mixture of **1** and $^{13}CO_2$ (1:2) at 298 K in [D$_8$]toluene (*). Ψ denotes peaks corresponding to **3**.

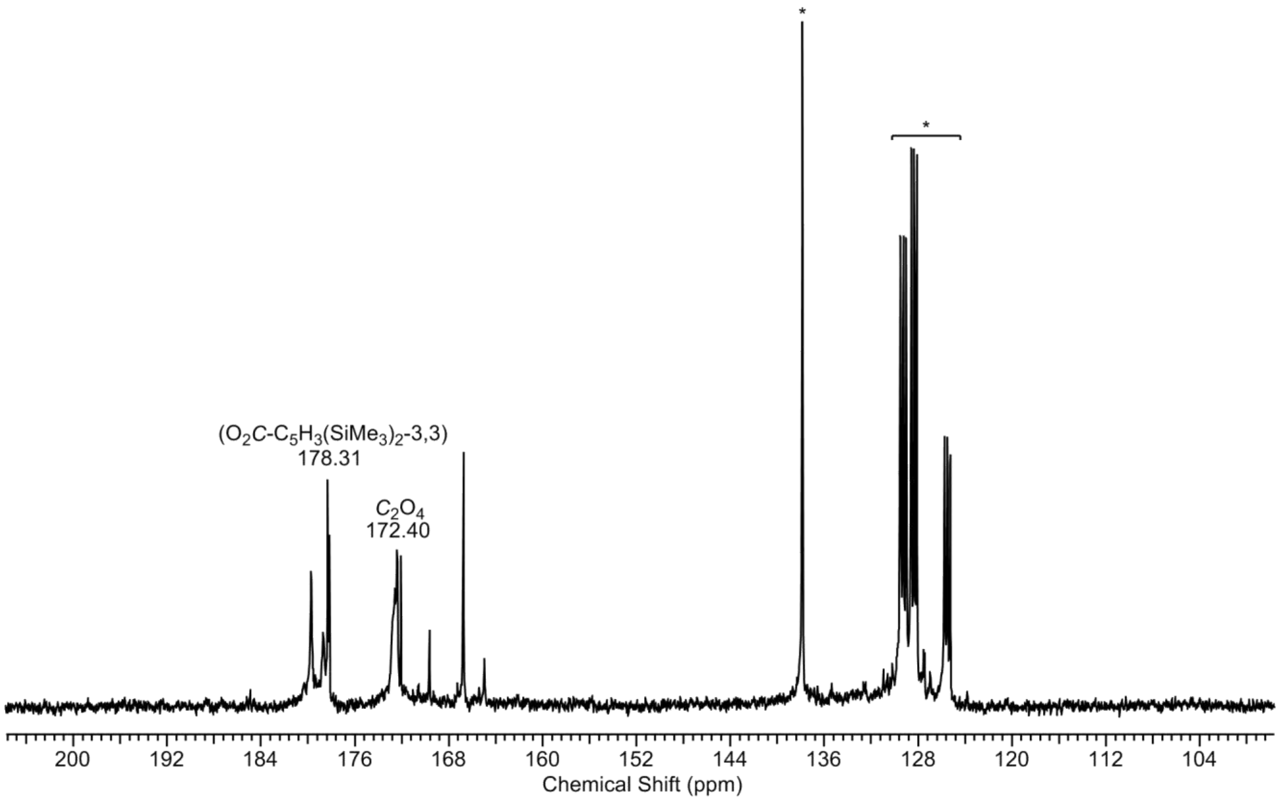

Figure S16. ^{13}C NMR spectrum of the reaction mixture of **1** and $^{13}CO_2$ (1:2) at 298 K in [D_8]toluene (*).

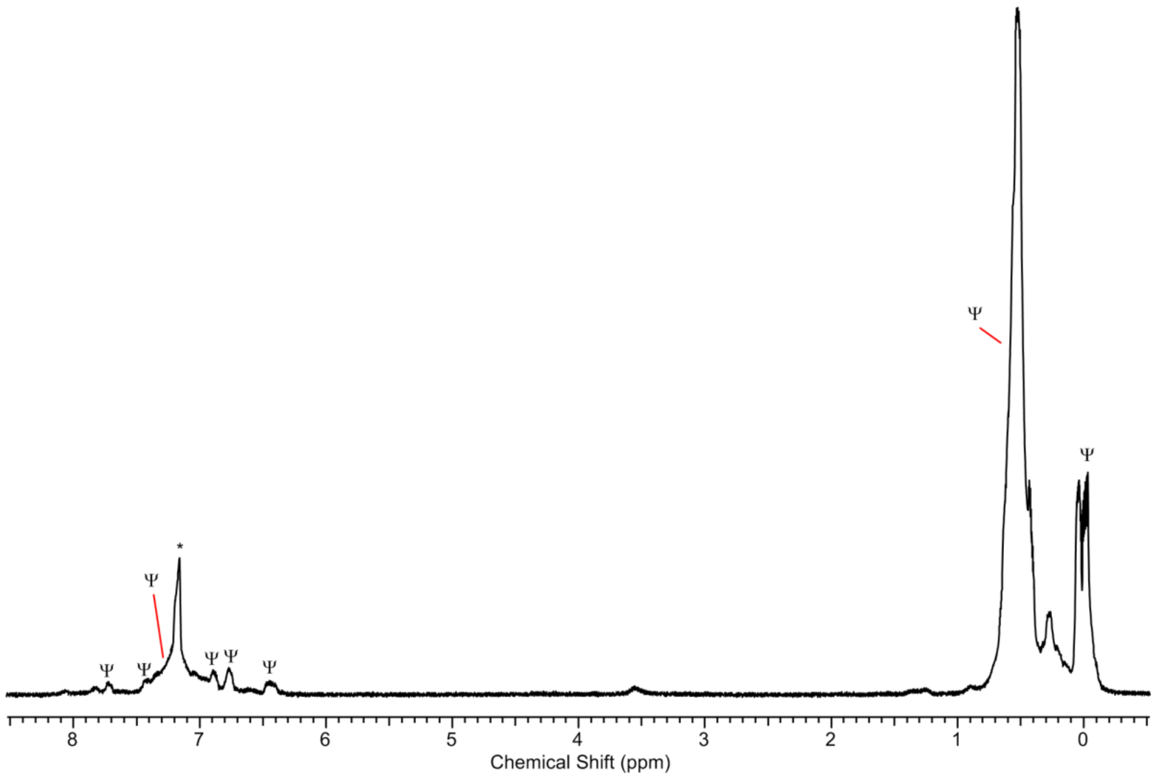

Figure S17. ^1H NMR spectrum of the reaction mixture of **1** and supercritical CO_2 in [D_6]benzene (*) at 298 K. Ψ denotes peaks corresponding to **3**.

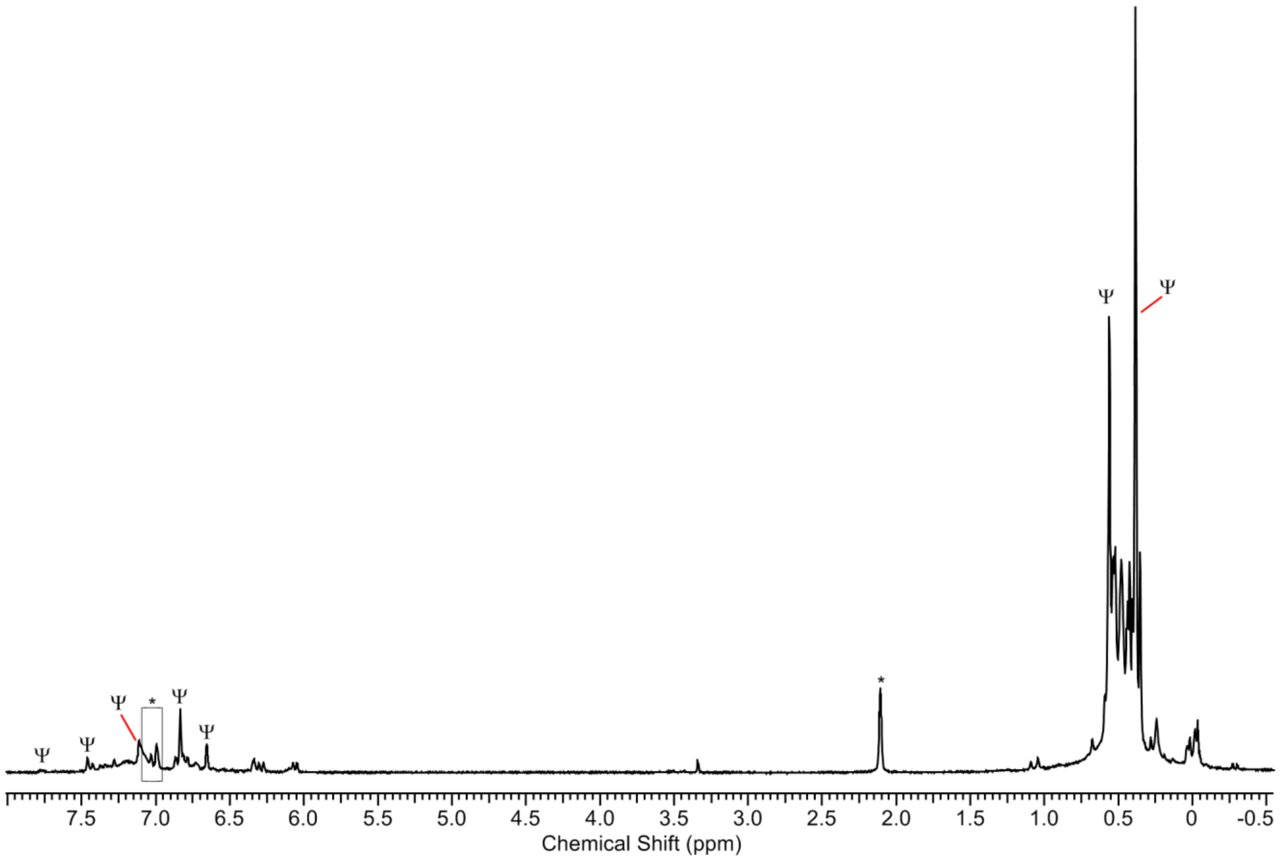

Figure S18. ^{1}H NMR spectrum of the reaction mixture of **1** and CO_2 (1:1) in [D_6]benzene (*) at 298 K. Ψ denotes peaks corresponding to **3**.

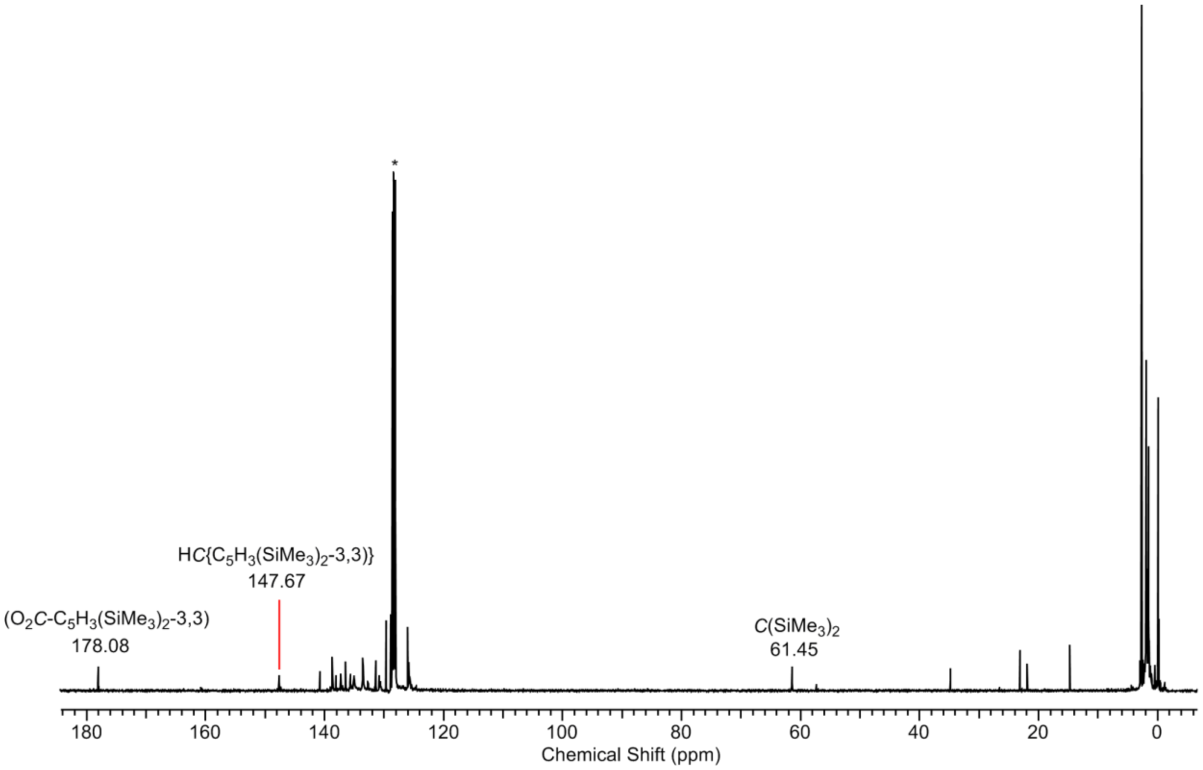

Figure S19. ^{13}C NMR spectrum of the reaction mixture of **2** and CO_2 in [D_6]benzene (*) at 298 K.

4. Crystallographic details

The crystal data for compounds **2**, **2b·2C_6H_{14}** and **3·2C_7H_8** are compiled in Table S1. Crystals were examined using Supernova Agilent equipped with CCD area detector and mirror-monochromated Mo Kα radiation (λ = 0.71073 Å). Intensities were integrated from data recorded on 1° frames by ω rotation. Cell parameters were refined from the observed positions of all strong reflections in each data set. A Gaussian grid face-indexed absorption correction with a beam profile correction was applied.[4] The structures were solved variously by direct and heavy atom methods using SHELXS[5] or SIR2004,[6] and were refined by full-matrix least-squares on all unique F^2 values,[5b] with anisotropic displacement parameters for all non-hydrogen atoms, and with constrained riding hydrogen geometries; U_{iso}(H) was set at 1.2 (1.5 for methyl groups) times U_{eq} of the parent atom. The largest features in final difference syntheses were close to heavy atoms and were of no chemical significance. CrysAlis^{Pro} was used for control and integration;[4] SHELX[5a] and SIR2004[6] were employed through OLEX2[7] for structure solution and refinement. ORTEP-3[8] and POV-Ray[9] were employed for molecular graphics. Highly disordered solvent molecules of crystallization in **2b** could not be modelled and were treated with the Platon SQUEEZE procedure.[10] CCDC 1497255 (**2**), 1497256 (**2b**) and 1497257 (**3·2C_7H_8**) contain the supplementary crystal data for this article. These data can be obtained free of charge from the Cambridge Crystallographic Data Centre *via* www.ccdc.cam.ac.uk/data_request/cif.

	**2**	**2b**	**3·2C$_7$H$_8$**
Formula	$C_{67}H_{126}S_2Si_{12}Th_2$	$C_{79}H_{154}S_2Si_{12}Th_2$	$C_{84}H_{142}O_8Si_{12}Th_2$
Fw	1796.95	1969.29	2081.13
cryst size, mm	0.18 x 0.11 x 0.05	0.18 x 0.16 x 0.08	0.20 x 0.16 x 0.11
crystal system	monoclinic	monoclinic	triclinic
space group	$P2_1/c$	$P2_1/n$	$P{-}1$
a, Å	14.1103(7)	28.4999(10)	11.6011(5)
b, Å	26.5761(11)	11.7361(3)	12.8156(6)
c, Å	23.5239(9)	31.0120(13)	17.6608(8)
α, °	90	90	78.707(4)
β, °	100.464(5)	116.404(5)	87.869(4)
γ, °	90	90	83.436(4)
V, Å^3	8674.7(7)	9290.8(7)	2557.7(2)
Z	4	4	1
ρ_{calcd}, g cm^3	1.376	1.397	1.351
μ, mm^{-1}	3.672	3.428	3.090
$F(000)$	3632	3632	1058
no. of reflections (unique)	28157(15724)	16942(16942)	18669(9326)
S[a]	0.99	1.04	1.05
R_1[b](wR_2)[c] ($F^2 > 2\sigma(F^2)$)	0.0505(0.0721)	0.0620(0.1187)	0.0326(0.0602)
R_{int}	0.050	0.088	0.036
min., max. diff map, e Å^{-3}	–1.40/1.32	–1.10/1.52	−0.66, 0.92

Table S1. Crystallographic data for **2**, **2b** and **3·2C$_7$H$_8$**. [a] $S(F^2) = [\Sigma w(Fo^2 - Fc^2)^2/(n + r - p)]^{½}$; [b] $R1(F) = \Sigma(|Fo| - |Fc|)/\Sigma|Fo|$; [c] $wR2(F^2) = [\Sigma w(Fo^2 - Fc^2)^2/\Sigma wFo^4]^{½}$.

5. Computational details

*5.1. Calculations on Optimized Structures of Models of **2** and **3***

Geometry optimisations were performed using version 6.6 of the TURBOMOLE software package[11] at the density functional level of theory, using the B3LYP hybrid-GGA exchange correlation functional.[12,13] Pople-style basis sets of 6-31G** quality[14] were used for light atoms and the Stuttgart-Dresden-Bonn relative effective core potential and corresponding basis set[15,16] was used for thorium. Geometry optimisation was followed by analytical vibrational frequency analysis to confirm the structures to be energetic minima. Subsequent single point energy calculations were performed in which the SARC all-electron basis set,[17] of TZVP quality, was applied to the thorium centre: in these all electron calculations, relativistic effects were included via application of the 2nd order Douglas-Kroll-Hess (DKH) Hamiltonian. Densities obtained from these calculations were subsequently analysed with the AIMAll[18] and Multiwfn[19] codes.

Structural parameters of **3** were in good agreement with experiment, whereas there was some deviation in the calculated structure of the CS_2 bridge of **2** (Table S2). Inspection of the resultant molecular orbitals reveals the presence of weak σ- and π-type Th–S bonding interactions in **2**, whereas only π-type Th–O bonding interactions were found in **3** (Figure S20). Bond orders, obtained via the quantum theory of atoms in molecules (QTAIM)/Mayer approaches, were calculated to be 0.441-0.462/0.539-0.713 for the Th–S bonds of **2** and 0.294-0.334/0.198-0.216 for the Th–O bonds of **3**. Th contributions to the MOs shown in Figure S20 were estimated via QTAIM and Hirshfeld partitioning of the molecular spaces. Both methods predicted small (~1-4%) Th contributions, with Hirshfeld-derived values being consistently larger (Table S4).

Optimized structures

2:
B3LYP/6-31G** Energy = -7712.652932872 a.u.

Th	-2.3109501	2.6134469	-1.5706763
Th	1.7144556	-2.2407206	1.2407875
Si	2.3567527	-3.0047485	5.8805027
Si	2.3136417	-3.3597930	-2.9788235
Si	3.0192138	-6.5452883	2.2024111
Si	6.2900311	-2.7897631	1.0372127
Si	-3.1796244	6.3606549	-3.8701945
Si	-2.1098283	-4.1029671	2.6160610
Si	-3.0249153	-1.0338345	-4.1947081
Si	1.2638907	2.7149003	-4.2330213
Si	3.7279025	1.4393039	2.8458502
Si	-3.7189245	1.8693581	2.6150098
Si	0.3736859	5.5281032	0.5475819
C	3.5565253	2.3564550	1.2011736
C	1.5233361	4.5841780	-4.3919811
C	-2.0952180	-4.0061449	4.5093714
C	-3.2911570	-2.2656532	-2.7842632
C	-3.1114063	-2.6493389	1.9338912
C	0.3538321	4.6065222	2.1941749
C	3.7590208	-7.2727462	0.6135561
C	-4.4372680	5.4282641	-4.9352770
C	-1.7176784	6.8554989	-4.9666512
C	6.5822256	-2.6751753	2.9060388
C	2.5339957	2.0163195	-3.0265521
C	-2.5673379	5.4282056	-2.3393697
C	1.9961249	5.2996990	-0.3913546
C	7.4454747	-1.5319778	0.2083942
C	-4.0632366	3.6257679	3.2443921
C	1.5745429	-4.7192409	6.1123287
C	1.5737073	1.9594548	-5.9465748
C	-3.9068552	1.7931704	0.7313496
C	0.5620340	-4.0827535	-3.0034793
C	0.1656325	7.3811774	0.9075334
C	2.0637740	-7.9384709	3.0706028
C	4.2095019	-3.1661685	6.2479605
C	4.4209811	-6.0196646	3.3596402
C	6.8166985	-4.5179174	0.4615014
C	-4.0144342	7.9365277	-3.2281054
C	3.5027511	-4.7433257	-3.5014995
C	-3.0185276	-5.6969444	2.1270369
C	-4.6905164	-0.7827888	-5.0635153
C	-1.8211717	-1.8004011	-5.4412797
C	-3.3320435	5.2252047	-1.1484919
C	1.5864448	-1.8963539	7.2170010
C	3.1460787	2.6027417	4.2297095
C	2.1159790	-2.0620853	4.2564666
C	2.3753523	-1.9785661	-4.2769525
C	3.1875471	-1.3095524	3.6981801
C	-3.7388711	0.6548818	-0.1339379
C	-2.0384497	1.3116282	3.2534780
C	0.9249802	-1.3544321	3.8709287
C	3.1130534	-1.2626563	-1.0212564
C	-1.1054404	5.0571490	-0.5339407
C	2.9583238	-2.6502685	-1.3508013
C	-4.7631735	2.6760118	0.0246146
C	-1.6951725	2.8367037	-4.3091508
C	-0.5149113	2.2240551	-3.7896426
C	-1.2028951	5.2979837	-1.9356900
C	-0.9096610	0.8911009	-3.4379988
C	3.8505093	-3.3383146	-0.4762249
C	0.7683670	-4.6959567	2.6482019
C	-0.0514613	-4.4679335	0.5425872
C	-2.7640370	1.9119712	-4.2625004
C	4.0565890	-1.1342902	0.0144198
C	1.7698744	-5.2036785	1.7705950
C	-2.2779742	0.6595241	-3.7545154
C	1.2243710	-5.0402417	0.4522605
C	-4.4946002	0.8604117	-1.3061382
C	4.5536028	-2.4332365	0.3698623
C	2.7106793	-0.1384947	3.0322678
C	1.2871165	-0.1878635	3.1661598
C	-2.4565859	4.9932660	-0.0654255
C	-0.3652189	-4.2302968	1.9241065
Si	-6.7266006	2.6679943	-2.1295533
C	-5.1388387	2.1412129	-1.2450349
C	-6.7284513	2.3972109	-4.0015154
C	-8.1181991	1.6114047	-1.3926933
C	-7.1134637	4.4882314	-1.7657507
C	-5.0625447	0.7285460	3.3142924
C	5.5677241	1.0881753	3.1419251
S	-0.0120515	1.5067546	0.0053329
C	0.3974046	-0.1593287	0.2096863
S	-0.4952782	-1.4640451	-0.3908783
H	-0.3562087	5.4353969	-2.5948725
H	-2.7597170	4.8457057	0.9628410
H	-4.4098533	5.2878468	-1.0801649
H	-3.7681665	2.0933387	-4.6276806
H	-1.7544152	3.8395615	-4.7086882
H	-0.2387970	0.1382483	-3.0399964
H	-5.1224403	3.6184397	0.4194297
H	-4.6009134	0.1508745	-2.1123790
H	-3.1518552	-0.2290064	0.0860886
H	4.2342664	-1.5453325	3.8409114
H	0.6002305	0.5650142	2.8085695
H	-0.0874087	-1.6193588	4.1534219
H	2.6031880	-0.4376221	-1.4973862
H	4.3987602	-0.1972611	0.4278406
H	4.0264499	-4.4060072	-0.4998451
H	-0.7098573	-4.2780275	-0.2942376
H	1.7039850	-5.3576007	-0.4636933
H	0.8322829	-4.7122671	3.7251039
H	-4.0252196	4.5120824	-5.3698796
H	-5.3332321	5.1611748	-4.3671889
H	-4.7588009	6.0688924	-5.7653152

H	-1.2687348	6.0031520	-5.4868215
H	-2.0613820	7.5570784	-5.7358936
H	-0.9278266	7.3540508	-4.3953842
H	-3.3195808	8.5364395	-2.6305705
H	-4.3686207	8.5588366	-4.0583688
H	-4.8783375	7.7063560	-2.5945266
H	0.1491238	7.9686514	-0.0172017
H	-0.7681343	7.5820294	1.4442295
H	0.9919854	7.7530604	1.5252883
H	2.8433811	5.5927787	0.2389202
H	2.1592020	4.2645215	-0.7011802
H	2.0223392	5.9362811	-1.2830541
H	1.1657074	4.9599765	2.8396373
H	-0.5868016	4.7780871	2.7307238
H	0.4753507	3.5298480	2.0497809
H	2.4711811	4.7575693	-4.9157800
H	0.7396881	5.0797457	-4.9728956
H	1.5985699	5.0802866	-3.4204384
H	2.5945899	2.1710579	-6.2869189
H	1.4465348	0.8716913	-5.9303077
H	0.8817946	2.3640611	-6.6938280
H	3.5161845	2.4643975	-3.2183282
H	2.2706671	2.2055454	-1.9820529
H	2.6434784	0.9356945	-3.1557770
H	-2.3926948	-2.3858371	-2.1748640
H	-4.1094719	-1.9836871	-2.1147644
H	-3.5418196	-3.2427123	-3.2152747
H	-0.8374935	-1.9779449	-4.9950289
H	-2.1998810	-2.7624755	-5.8062002
H	-1.6796043	-1.1439599	-6.3068183
H	-4.6368465	-0.0407897	-5.8668832
H	-5.0086261	-1.7312914	-5.5122505
H	-5.4794082	-0.4761042	-4.3696617
H	-6.4062108	5.1762349	-2.2399985
H	-8.1107059	4.7343885	-2.1490147
H	-7.1207280	4.7001745	-0.6909044
H	-5.9527706	2.9686732	-4.5180655
H	-6.6020000	1.3428961	-4.2635611
H	-7.6953486	2.7163821	-4.4093071
H	-8.1936873	1.7538813	-0.3091771
H	-9.0874587	1.8714655	-1.8345601
H	-7.9447588	0.5446332	-1.5723771
H	-1.2482561	2.0095874	2.9643194
H	-2.0515237	1.2470001	4.3479371
H	-1.7676332	0.3262643	2.8636808
H	-5.0454685	0.7297609	4.4106982
H	-6.0605033	1.0476594	2.9936297
H	-4.9206081	-0.3036982	2.9780406
H	-3.2522359	4.3248740	3.0150806
H	-4.9946188	4.0427957	2.8461441
H	-4.1629849	3.6000647	4.3360561
H	-3.1704306	-2.6789235	0.8410742
H	-4.1354129	-2.6841888	2.3254102

H	-2.6731551	-1.6875462	2.2159057
H	-2.4999289	-6.5810757	2.5138095
H	-4.0427513	-5.7081274	2.5190492
H	-3.0772330	-5.8012534	1.0377940
H	-1.5874878	-3.1140726	4.8900891
H	-3.1241420	-3.9832807	4.8875432
H	-1.6071670	-4.8835815	4.9471366
H	2.7251740	-8.7775313	3.3183751
H	1.6041756	-7.5906823	4.0024966
H	1.2600553	-8.3212841	2.4319032
H	5.0288333	-5.2187113	2.9327092
H	4.0364420	-5.6697347	4.3223016
H	5.0837794	-6.8698587	3.5612117
H	4.4137670	-8.1166918	0.8612950
H	2.9738214	-7.6522686	-0.0496027
H	4.3587840	-6.5544513	0.0456993
H	0.3070322	-4.3680818	-4.0315786
H	0.4669764	-4.9784448	-2.3828054
H	-0.1820319	-3.3568480	-2.6642319
H	3.3749366	-1.5361117	-4.3447951
H	2.1110797	-2.3669814	-5.2676708
H	1.6693513	-1.1743407	-4.0417740
H	4.5325909	-4.3782876	-3.5786339
H	3.5001006	-5.5762796	-2.7889217
H	3.2155361	-5.1501286	-4.4783455
H	4.1223525	1.8897164	0.3889547
H	2.5109412	2.4112414	0.8857868
H	3.9344184	3.3794175	1.3152802
H	3.7218988	3.5363790	4.2308699
H	2.0882984	2.8621676	4.1177815
H	3.2677607	2.1319244	5.2116732
H	6.1221147	2.0343700	3.1488825
H	5.7424936	0.6000504	4.1067680
H	6.0078354	0.4607503	2.3607601
H	2.0161596	-0.8892281	7.1904286
H	0.5042207	-1.7951671	7.0772970
H	1.7550164	-2.3102613	8.2184934
H	1.7607204	-5.0340095	7.1468733
H	0.4893798	-4.7088968	5.9710285
H	1.9972957	-5.4881334	5.4595041
H	4.6997058	-2.1876481	6.2805003
H	4.3517520	-3.6391880	7.2268674
H	4.7362486	-3.7789122	5.5099507
H	7.8617334	-4.6988425	0.7396076
H	6.2187775	-5.3177440	0.9083634
H	6.7425213	-4.6126360	-0.6271641
H	8.4880778	-1.7133066	0.4958564
H	7.3805524	-1.5990033	-0.8831058
H	7.2007733	-0.5017041	0.4890124
H	7.6099681	-2.9926087	3.1222185
H	6.4751806	-1.6524213	3.2773743
H	5.9130608	-3.3168116	3.4846686

3:

B3LYP/6-31G** Energy = -7632.563755332 a.u.

Th	0.6392406	-2.5575281	1.9356154
Si	3.0416871	-3.3618663	-1.5341476
Si	1.0065994	-6.2996353	4.1009189
Si	-3.5197634	-3.5515400	1.6961531
Si	4.0050262	-0.5249986	3.5558127
Si	-3.4886794	-0.6692931	8.1739320
Si	-1.7658196	2.1019133	7.5438368
O	-0.2552200	-1.7802697	-0.2488938
O	-0.7915332	-0.0461488	-1.5978348
O	0.8360439	-2.0782837	4.3072355
O	-1.0478613	-1.4070919	3.3717098
C	2.9948869	-2.9605266	0.3090165
C	0.0947532	-5.2690625	2.8120474
C	-1.1063960	-4.5450122	3.0701229
C	3.1791925	-1.6836781	0.9237746
C	-1.7476927	-4.1683865	1.8545818
C	3.3006400	-3.2251083	2.5887534
C	3.0855235	-3.9095530	1.3738623
C	3.3733469	-1.8188795	2.3291023
C	-0.3348194	-0.5508324	-0.5445622
C	-0.9077739	-4.6685093	0.8099683
C	0.1947675	-5.3346086	1.3851190
C	-0.3074248	-1.4898301	4.4098388
C	-1.8491341	-0.1915613	5.9401802
C	-0.7244270	-0.9444368	5.7064574
C	-2.0489961	2.6560704	9.3333654
C	-1.9231744	0.1687832	7.3769888
C	-0.0072183	-1.1182080	6.9561146
C	1.2515936	-5.3203856	5.7022329
C	-0.6967276	-0.4801670	7.9405647
C	1.4944242	-4.2693013	-2.1423558
C	4.3490522	-1.3937941	5.2026686
C	-3.0057347	2.9837522	6.4173142
C	2.6746324	-6.9269950	3.4553199
C	4.5497176	-4.4746032	-1.8301335
C	3.2512055	-1.7694189	-2.5344590
C	2.8278708	0.9239831	3.8591717
C	-4.1724771	-3.0801640	3.4080812
C	-3.6576957	-2.0678609	0.5320970
C	-0.0731234	-7.8138391	4.4794157
C	-4.5602621	-4.9711346	0.9886941
C	5.6285864	0.1591066	2.8499366
C	-0.0206390	2.6064583	7.0217643
C	-3.3451372	-0.6810508	10.0624283
C	-5.0833019	0.2232050	7.6763368
C	-3.5753838	-2.4596486	7.5720944
O	0.0814717	1.6700764	0.2370673
O	0.6612196	-0.0655463	1.5647047
C	0.1821077	0.4393725	0.5218061
Th	-0.7882117	2.4492859	-1.9532673
Si	-3.3544493	3.1251036	1.4277618
Si	-1.1929082	6.2297297	-4.0550999
Si	3.3322940	3.5047028	-1.6250193
Si	-4.0394097	0.3901601	-3.7613657
Si	3.7405047	1.0622716	-7.9818399
Si	2.3195490	-1.9021819	-7.5209501
O	-0.8597143	1.9564628	-4.3288565
O	1.0062214	1.3682271	-3.3054532
C	-3.2187330	2.7674449	-0.4200588
C	-0.2915968	5.1913647	-2.7652201
C	0.9298275	4.4994919	-3.0144241
C	-3.3533103	1.5032011	-1.0726485
C	1.5573949	4.1124973	-1.7948596
C	-3.4338336	3.0824074	-2.7039319
C	-3.2827093	3.7405779	-1.4650158
C	-3.4900694	1.6692240	-2.4810787
C	0.6877649	4.5731729	-0.7568648
C	-0.4198224	5.2250001	-1.3393397
C	0.3192034	1.4352230	-4.3811887
C	2.0658415	0.3603968	-5.8600455
C	0.8444261	0.9577272	-5.6645598
C	1.9795886	-2.3681940	-9.3249056
C	2.2402370	0.0302779	-7.2949994
C	0.1571022	1.0499471	-6.9393371
C	-1.3720615	5.2771257	-5.6812941
C	0.9594430	0.5165907	-7.9003107
C	-1.8510463	4.0477787	2.1184678
C	-4.2744651	1.2753062	-5.4187019
C	4.0072290	-2.5956838	-7.0113908
C	-2.8909505	6.8106037	-3.4453217
C	-4.8940332	4.2045615	1.6799909
C	-3.5795621	1.5059245	2.3815842
C	-2.8433801	-1.0557364	-3.9940716
C	4.0697061	3.2760674	-3.3535614
C	3.4654216	1.8786365	-0.6694992
C	-0.1361729	7.7734806	-4.3746457
C	4.3078017	4.8354688	-0.6897725
C	-5.7059822	-0.3008582	-3.1712290
C	0.9827423	-2.6827807	-6.4363510
C	4.1621938	0.5509254	-9.7566458
C	5.2698045	0.8464175	-6.8875732
C	3.2559219	2.8903165	-7.9772748
H	2.7871305	0.1512649	-5.0794942
H	0.7262433	0.4467073	-8.9566489
H	-0.8263437	1.4825302	-7.0743383
H	1.3424297	4.3153833	-4.0005705
H	0.8511672	4.4476361	0.3071678
H	-1.2189575	5.7043688	-0.7882816
H	-3.3744358	0.5473985	-0.5635721
H	-3.5225465	3.5670148	-3.6692856
H	-3.2551221	4.8130559	-1.3292309
H	3.1960198	-0.7412624	0.3896083
H	3.4200846	-3.6880055	3.5613976
H	3.0331998	-4.9843026	1.2661895
H	-1.0892398	-4.5616894	-0.2531835
H	0.9727180	-5.8422062	0.8293595
H	-1.4955992	-4.3303329	4.0595356
H	-0.4128483	-0.4292874	8.9856027

H	0.9192461	-1.6694543	7.0579503
H	-2.5705353	0.0989569	5.1859338
H	0.8661451	7.5027898	-4.7244944
H	-0.5941378	8.4167739	-5.1356490
H	-0.0164826	8.3675141	-3.4617726
H	-2.8239940	7.2944460	-2.4639959
H	-3.2976770	7.5524259	-4.1428752
H	-3.6194235	5.9977659	-3.3715001
H	-0.3921770	5.0064315	-6.0906937
H	-1.9367742	4.3483885	-5.5542567
H	-1.8839202	5.8895503	-6.4330363
H	5.1027987	2.9150103	-3.2876235
H	3.4885890	2.5426649	-3.9222403
H	4.0826808	4.2151890	-3.9177948
H	5.3613541	4.5512005	-0.5820088
H	4.2703896	5.7965677	-1.2142995
H	3.9025870	4.9928810	0.3162136
H	4.5172036	1.6153579	-0.5042088
H	2.9755809	1.9444573	0.3075120
H	2.9991567	1.0657107	-1.2348949
H	-0.9389246	3.4526775	2.0084475
H	-1.6934874	5.0081519	1.6147117
H	-1.9909904	4.2593005	3.1854679
H	-2.6700368	0.8954665	2.3983822
H	-3.8438792	1.7277670	3.4226017
H	-4.3888798	0.8986899	1.9627302
H	-4.8137557	5.1515882	1.1343337
H	-5.7962927	3.6957595	1.3232122
H	-5.0391162	4.4446020	2.7400070
H	-1.9068402	-0.7239371	-4.4515054
H	-2.5910582	-1.5381348	-3.0454725
H	-3.2942415	-1.8090259	-4.6517271
H	-3.3298588	1.7140851	-5.7568612
H	-4.6124050	0.5708799	-6.1876776
H	-5.0197275	2.0757489	-5.3536377
H	-5.6020776	-0.8194828	-2.2114177
H	-6.4440989	0.4977277	-3.0378616
H	-6.1131360	-1.0186126	-3.8934245
H	4.1228717	3.5080893	-8.2397423
H	2.4595321	3.1047751	-8.6960299
H	2.9086484	3.2073281	-6.9887394
H	4.5814719	-0.4587976	-9.8143265
H	3.2889793	0.5930143	-10.4163998
H	4.9123923	1.2399992	-10.1627684
H	6.1051437	1.4115837	-7.3184567
H	5.0974810	1.2385307	-5.8797008
H	5.5904755	-0.1949771	-6.7956445
H	2.0459465	-3.4573430	-9.4349697
H	0.9715122	-2.0698680	-9.6307909
H	2.6912255	-1.9230509	-10.0260408
H	4.8089736	-2.2927042	-7.6927729
H	4.2914652	-2.2898525	-5.9988707
H	3.9660997	-3.6915242	-7.0224848
H	1.2248119	-2.6099890	-5.3716157
H	0.0122125	-2.2002459	-6.5894736
H	0.8705163	-3.7452095	-6.6832908
H	3.4087428	-2.0225488	-3.5900941
H	2.3708588	-1.1196520	-2.4838583
H	4.1198170	-1.1917844	-2.2005658
H	1.3391287	-5.2168163	-1.6143445
H	0.6000392	-3.6530729	-2.0072728
H	1.5858711	-4.5023798	-3.2102009
H	5.4755129	-3.9768188	-1.5213146
H	4.4753542	-5.4099972	-1.2639295
H	4.6454227	-4.7369023	-2.8905236
H	6.3560185	-0.6415640	2.6765578
H	5.4618736	0.6680844	1.8938857
H	6.0823600	0.8838959	3.5363962
H	4.7413718	-0.6825376	5.9388214
H	3.4283997	-1.8242105	5.6105046
H	5.0847126	-2.1985624	5.0955068
H	2.5072042	1.3988177	2.9277771
H	1.9277033	0.5945051	4.3865451
H	3.3241574	1.6816854	4.4777663
H	3.0888711	-7.6604784	4.1572253
H	2.5694304	-7.4311830	2.4876847
H	3.4149615	-6.1295592	3.3413068
H	1.8297222	-4.4057230	5.5395994
H	0.2891288	-5.0224082	6.1335225
H	1.7726568	-5.9290307	6.4507107
H	0.3924705	-8.4527433	5.2394865
H	-1.0591665	-7.5150290	4.8520157
H	-0.2313108	-8.4218535	3.5817389
H	-3.1825381	-2.2658105	-0.4344465
H	-4.7092101	-1.8163954	0.3482733
H	-3.1760038	-1.1904264	0.9741425
H	-3.5471667	-2.2981083	3.8500345
H	-5.1997139	-2.7033953	3.3421874
H	-4.1786981	-3.9403330	4.0870993
H	-4.5126371	-5.8559753	1.6329429
H	-5.6133350	-4.6804472	0.8934735
H	-4.2061194	-5.2673748	-0.0050881
H	-2.6130462	-2.9662700	7.6974722
H	-4.3236376	-3.0147617	8.1501641
H	-3.8536271	-2.5234069	6.5156979
H	-2.4763407	-1.2593890	10.3937458
H	-3.2694146	0.3201944	10.4953199
H	-4.2363697	-1.1580170	10.4879293
H	-5.1771871	0.3240173	6.5898167
H	-5.9466784	-0.3554492	8.0260045
H	-5.1599430	1.2225130	8.1171032
H	-1.8018921	3.7206617	9.4239832
H	-3.0896699	2.5346659	9.6512779
H	-1.4133971	2.1121685	10.0402146
H	-2.7971026	2.7729527	5.3633052
H	-4.0451862	2.7099065	6.6190119
H	-2.9165780	4.0677628	6.5582620
H	0.7334229	2.2646579	7.7369331
H	0.0483918	3.6988768	6.9568707
H	0.2419326	2.1975094	6.0409598

	2		3	
	**B3LYP/6-31G****	**Experimental**	**B3LYP/6-31G****	**Experimental**
$\boldsymbol{r}_{\textbf{Th-L}}$ **(Å)**	Th_1-C_1: 2.670<br>Th_1-S_1: 2.854<br>Th_2-S_2: 2.999	Th_1-C_1: 2.826<br>Th_1-S_1: 2.886<br>Th_2-S_2: 2.916	Th-O_3: 2.484<br>Th-O_4:2.521	Th-O_3: 2.445<br>Th-O_4: 2.505
$\boldsymbol{r}_{\textbf{Th-Cp}}$ **(Å)**	Th_1: 2.608<br>Th_2: 2.678	Th_1: 2.596<br>Th_2: 2.579	2.598	2.552

Table S2. Comparison of key in PBE0/SVP optimised and experimental structural parameters of **2** and **3** (**2** : L = C, S, **3**: L = O). Values are averaged over both Th centres. All values are in Å.

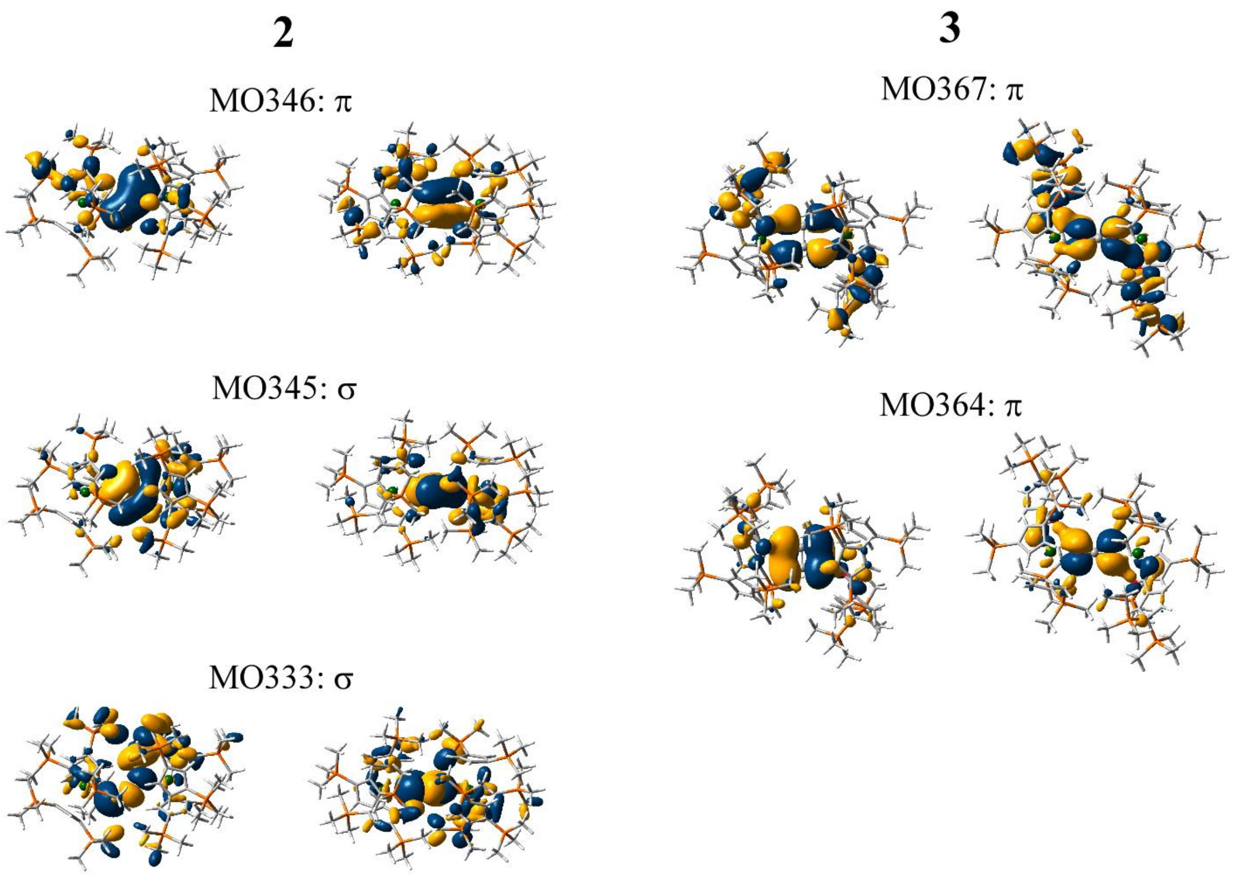

Figure S20. Selected B3LYP/6-31G** MOs of **2** and **3** exhibiting Th σ- and π-bonding character. Orbitals rendered at an isosurface of 0.015 a.u.

Bond Order	2	3
**QTAIM**	Th_1-C_1: 0.395<br>Th_1-S_1: 0.462<br>Th_2-S_2: 0.441	Th-O_3: 0.334<br>Th-O_4: 0.294
**Mayer**	Th_1-C_1: 0.210<br>Th_1-S_1: 0.713<br>Th_2-S_2: 0.539	Th-O_3: 0.216<br>Th-O_4: 0.198

Table S3. Bond order analyses of **2** and **3**.

	2		3	
	**MO**	**Th contribution (%)**	**MO**	**Th contribution (%)**
**QTAIM**	346<br>345<br>333	Th_1: 1.7 Th_2: 0.5<br>Th_1: 4.2 Th_2: 1.0<br>Th_1: 1.1 Th_2: 1.5	367<br>364	1.0<br>1.4
**Hirshfeld**	346<br>345<br>333	Th_1: 4.1 Th_2: 1.4<br>Th_1: 7.4 Th_2: 2.2<br>Th_1: 2.2 Th_2: 3.0	367<br>364	2.6<br>3.4

Table S4. Percentage thorium contributions to bonding MOs (See Figure S17), derived from QTAIM- and Hirshfeld-partitioning of the molecular spaces of **2** and **3.**

	ρ_{BCP}	$\nabla^2\rho_{BCP}$	H_{BCP}	δ
**2**	Th_1-C_1: 0.052<br>Th_1-S_1: 0.050<br>Th_2-S_2: 0.043	Th_1-C_1: 0.085<br>Th_1-S_1: 0.079<br>Th_2-S_2: 0.050	Th_1-C_1: -0.009<br>Th_1-S_1: -0.009<br>Th_2-S_2: -0.008	Th_1-C_1: 0.395<br>Th_1-S_1: 0.462<br>Th_2-S_2: 0.441
**3**	Th-O_3: 0.057<br>Th-O_4: 0.053	Th-O_3: 0.171<br>Th-O_4: 0.157	Th-O_3: -0.007<br>Th-O_4: -0.005	Th-O_3: 0.334<br>Th-O_4: 0.294

Table S5. QTAIM analysis of the M-L bond in of **2** and **3**. ρ_{BCP} = magnitude of the electron density at the M-L bond critical point, $\nabla^2\rho_{BCP}$ = Laplacian of $\rho_{BCP.}$ H_{BCP} = energy density at the BCP, δ = delocalisation index, ε_{BCP} = ellipticity of the M-L bond. All values are in a.u.

5.1. Calculations on Optimized Structures of Reaction Pathway to give ***3***

All calculations were carried out with the Gaussian 09 suite of programs.[20] Thorium atoms were treated with a 5f-in-core effective core potential to describe their +4 oxidation state, associated with its adapted basis set.[21] Carbon, oxygen and hydrogen atoms were described with a 6-31G(d,p) double-ζ basis set.[22] Calculations were carried out at the DFT level of theory with the hybrid functional B3PW91.[12,23] Geometries were optimized without any symmetry restriction and the nature of the extrema was verified by analytical frequency calculations. The calculation of electronic energies and enthalpies of the extrema of the potential energy surface (minima and transition states) were performed at the same level of theory as the geometry optimizations. IRC calculations were performed to confirm the connections of the optimized transition states. The electronic charges (at the DFT level) were computed using the natural population analysis (NPA) technique.[24] Despite our considerable efforts to locate, classical oxalate formation TS from a $\{Th_2(\mu\text{-}\kappa^1\text{:}\kappa^2\text{-}CO_2)\}$ complex were not successful as it always led to the reported TS that involves a dioxocarbene intermediate formation.

Cartesian coordinates of all optimized structures

CO_2

E = -188.504053
H = -188.489902

C	0.033505	0.000000	0.000584
O	-0.012709	0.000000	1.168047
O	0.028063	0.000000	-1.167778

Complex C1

E = -2897.877942
H = -2896.006507

C	-0.134367	0.022186	0.079641
C	-0.368235	-0.029181	1.484343
C	0.925553	-0.067565	2.081427
C	1.905690	-0.036151	1.069706
C	1.261049	0.047467	-0.207310
Th	0.803653	-2.595297	0.888645
O	1.286410	-2.910490	3.100782
C	1.589722	-2.891567	4.377042
Th	2.481547	-3.055394	6.750558
C	0.701247	-1.301494	8.247074
C	0.616041	-2.462311	9.061096
C	1.841225	-2.684435	9.751032
C	2.720790	-1.660750	9.308193
C	2.039036	-0.824180	8.396513
Si	-1.944284	0.565374	2.352738
C	-3.354523	0.777512	1.099996
Si	-0.761816	-0.216199	7.776327
C	-1.157449	0.837872	9.309625
Si	2.004131	-3.623766	11.383659
C	3.548148	-4.713652	11.514748
Si	1.933853	1.021681	-1.692330
C	3.622801	0.501557	-2.385523
C	-1.522658	-3.888137	1.800923
C	-0.962586	-4.893168	0.945490
C	-1.122080	-4.385581	-0.373863
C	-1.773915	-3.120972	-0.372591
C	-2.005109	-2.831032	1.011364
Si	-0.860867	-6.758355	1.275688
C	0.572986	-7.345797	2.358829
Si	-2.899919	-2.576628	-1.804795
C	-2.741474	-3.823423	-3.225372
C	3.121961	-4.237014	0.734984
C	3.633723	-2.986414	0.264056
C	2.994077	-2.774882	-0.989195
C	2.140216	-3.862323	-1.325760
C	2.231729	-4.765820	-0.218341
Si	5.367380	-2.307329	0.652249
C	5.499234	-0.547851	1.345635
Si	1.794943	-4.260938	-3.144928
C	1.026896	-2.777034	-4.036098

C	-2.494350	-0.557379	3.773883
C	-1.541342	2.297634	3.014514
C	2.135366	2.793304	-1.036552
C	0.685251	1.062024	-3.114980
C	-2.666437	-0.838688	-2.521047
C	-4.674157	-2.699181	-1.139103
C	0.764894	-5.827211	-3.420556
C	3.491230	-4.597526	-3.932656
C	-0.738235	-7.675011	-0.379934
C	-2.495716	-7.256382	2.098558
C	6.247706	-3.478053	1.849520
C	6.317674	-2.342941	-0.992348
O	0.821762	-2.244594	5.179073
C	3.087204	-5.908031	6.232613
C	3.093892	-5.716339	7.652256
C	1.781076	-5.452934	8.084074
C	0.908495	-5.414394	6.956089
C	1.744342	-5.693153	5.830117
Si	4.264477	-7.014073	5.248720
C	6.035569	-7.084989	5.915939
Si	-0.953913	-5.735198	6.927751
C	-1.173620	-7.619968	6.810535
C	3.549829	-8.769672	5.375767
C	4.327090	-6.524861	3.426806
C	4.539474	-2.086142	5.052233
C	4.469610	-0.976378	5.954784
C	4.994429	-1.466563	7.178847
C	5.382912	-2.827490	7.084082
C	5.076620	-3.200491	5.728776
Si	4.521388	0.884092	5.599695
C	4.407445	1.895736	7.204618
Si	6.742704	-3.467463	8.242944
C	6.984423	-2.197932	9.636074
C	3.202400	1.519259	4.405712
C	6.236723	1.243576	4.863733
C	-1.708763	-4.945725	5.386511
C	-1.886157	-5.160508	8.472579
C	-0.297756	0.958116	6.370197
C	-2.301831	-1.242418	7.380895
C	0.472766	-4.698030	11.711074
C	2.097062	-2.307752	12.749999
C	6.527974	-5.156407	9.071742
C	8.336895	-3.526972	7.211278
H	1.121884	-0.122217	3.144266
H	-3.074449	-1.418584	3.429460
H	-3.123094	0.004748	4.471835
H	-1.629743	-0.936463	4.325479
H	-0.697283	2.278590	3.709102
H	-2.402989	2.727436	3.537562
H	-1.279389	2.965929	2.187725
H	-4.227253	1.202483	1.608210
H	-3.067716	1.472792	0.304317
H	-3.669731	-0.160849	0.636224
H	1.190643	3.164645	-0.627445
H	-0.912995	0.112240	-0.667671
H	2.451754	3.472178	-1.836318
H	2.882548	2.838335	-0.237852

H	-2.867315	-0.057776	-1.782364
H	-1.663761	-0.682860	-2.926421
H	0.531922	0.076750	-3.562849
H	1.056170	1.729655	-3.900632
H	-3.379987	-0.701618	-3.341723
H	-0.285237	1.446928	-2.788236
H	-5.397831	-2.487389	-1.934021
H	-4.873353	-3.704978	-0.756256
H	3.571786	-0.383098	-3.026910
H	4.355903	0.313235	-1.596126
H	4.007718	1.324336	-2.998652
H	-1.749480	-3.823309	-3.684341
H	-3.465928	-3.572560	-4.008057
H	-2.961024	-4.839994	-2.884222
H	1.725538	-1.934604	-4.040517
H	0.802139	-3.023199	-5.079345
H	-0.274144	-5.753600	-3.092201
H	3.987804	-5.440859	-3.441971
H	0.753662	-6.047196	-4.494222
H	4.149351	-3.727692	-3.844196
H	3.387577	-4.838491	-4.996614
H	1.222316	-6.686188	-2.919928
H	0.197414	-7.468445	-0.906542
H	-1.566360	-7.418250	-1.047424
H	-0.780241	-8.754429	-0.197008
H	-2.630208	-6.760268	3.063805
H	-2.529928	-8.338349	2.267309
H	-3.343808	-6.984402	1.462222
H	0.517811	-8.433136	2.482009
H	0.537475	-6.898184	3.354353
H	1.542047	-7.109961	1.910765
H	-1.591121	-3.936928	2.880212
H	3.395734	-4.711220	1.668151
H	3.200489	-1.940522	-1.646191
H	7.303165	-3.194371	1.926145
H	5.826285	-3.435760	2.856874
H	6.202868	-4.511413	1.493392
H	6.310380	-3.349860	-1.421272
H	5.884926	-1.661226	-1.730894
H	7.361312	-2.047754	-0.836646
H	4.991014	-0.446335	2.308110
H	5.109041	0.213494	0.663740
H	6.559445	-0.323469	1.509318
H	2.194047	1.344342	4.789700
H	1.391065	-5.778682	4.810275
H	7.023959	0.951602	5.566381
H	6.352088	2.312055	4.649378
H	6.399687	0.692046	3.933811
H	4.235262	-2.078260	4.013556
H	3.482642	-9.086858	6.421361
H	4.835951	-5.568633	3.284247
H	4.179177	-9.490208	4.841375
H	4.860830	-7.283118	2.843885
H	3.315038	-6.441730	3.024210
H	2.543081	-8.814540	4.949065
H	6.070345	-7.518399	6.919785
H	6.526359	-6.108683	5.942256

H	6.627538	-7.734879	5.261447
H	-1.518428	-3.869432	5.360669
H	-1.256715	-5.387425	4.493585
H	-2.789314	-5.116416	5.338517
H	0.534329	1.602596	6.673056
H	-1.383696	-5.461933	9.395968
H	-2.880136	-5.621946	8.473246
H	0.004668	0.392354	5.485737
H	-2.615328	-1.811594	8.262230
H	-1.138584	1.605649	6.103357
H	-2.128253	-1.942428	6.560760
H	-3.135818	-0.590515	7.100687
H	-1.420401	0.203296	10.162118
H	-0.293216	1.443596	9.600361
H	-1.997973	1.514874	9.118996
H	-0.279764	-3.054356	9.200510
H	3.734602	-1.516421	9.659233
H	2.442318	0.070415	7.941475
H	0.501627	-5.060607	12.744671
H	0.421177	-5.576679	11.060951
H	-0.451666	-4.125697	11.586984
H	1.201679	-1.678340	12.743241
H	2.182107	-2.770517	13.739477
H	2.962614	-1.652999	12.606714
H	3.611418	-5.157183	12.514510
H	4.458150	-4.127832	11.354773
H	3.530745	-5.530100	10.786649
H	3.959625	-5.824128	8.294399
H	5.129636	-0.856052	8.062565
H	5.103738	1.539926	7.970061
H	3.405049	1.915662	7.641340
H	8.541013	-2.551156	6.759632
H	6.408963	-5.964186	8.348302
H	8.263882	-4.258563	6.400183
H	9.194883	-3.802394	7.834547
H	7.430682	-5.369369	9.656349
H	6.083006	-2.073178	10.245079
H	7.271470	-1.215752	9.248526
H	7.784233	-2.538015	10.303113
H	-2.025877	-4.077266	8.490195
H	-4.857885	-1.992024	-0.324484
H	4.680162	2.932240	6.975060
H	-0.764314	-8.118089	7.695520
H	-0.656916	-8.023840	5.934346
H	-2.233837	-7.886028	6.732727
H	0.102453	-2.445156	-3.556170
H	3.279688	1.052781	3.420827
H	3.324488	2.599164	4.266044
H	2.974431	-0.023170	1.234438
H	1.477760	-5.312618	9.110646
H	-0.845928	-4.926453	-1.269513
H	-2.527811	-1.965942	1.390493
H	1.727206	-5.720501	-0.141408
H	5.272901	-4.164987	5.278710
H	5.680011	-5.175206	9.759726

Complex C2

E = -2897.866765
H = -2895.996180

C	1.268858	-2.353977	1.009992
C	1.886051	-1.095868	0.884726
C	2.010388	-0.775211	-0.508322
C	1.402702	-1.853406	-1.198655
C	0.935944	-2.850283	-0.287065
Th	-0.846144	-0.673088	0.136620
C	-2.229404	-0.792427	2.645038
C	-3.194043	-0.941049	1.626204
C	-3.073665	-2.248102	1.061644
C	-1.990235	-2.861093	1.748658
C	-1.468267	-1.991165	2.749024
Si	-4.513289	-3.148901	0.232487
C	-5.419750	-1.978685	-0.933270
Si	-0.553076	-2.534113	4.315906
C	0.036737	-4.332015	4.182606
Si	3.347307	0.317004	-1.286993
C	4.802026	-0.838725	-1.676873
Si	0.732370	-4.661090	-0.801459
C	-0.241297	-4.781008	-2.416003
O	-1.533297	-0.969242	-1.983911
C	-2.323496	-1.011313	-3.049642
O	-1.654348	-1.000278	-4.191367
Th	-1.703914	-0.927609	-6.472002
C	0.336136	0.853044	-5.753113
C	-0.643577	1.735346	-6.306812
C	-0.617677	1.488786	-7.708976
C	0.354811	0.506770	-8.043615
C	0.926660	0.109381	-6.790317
Si	-1.250809	3.355149	-5.534378
C	0.190935	4.067533	-4.528803
Si	1.270067	0.532029	-9.709085
C	3.090991	0.894168	-9.309420
C	-2.189515	-3.711680	-6.790558
C	-1.087390	-3.614922	-5.922397
C	0.060362	-3.196223	-6.668389
C	-0.402253	-3.041185	-8.008980
C	-1.780556	-3.375959	-8.120982
Si	1.873475	-3.589989	-6.296137
C	1.975211	-5.462505	-6.001272
Si	-2.496139	-4.181331	-9.686900
C	-1.420817	-3.803809	-11.200103
C	-3.844440	0.864447	-6.818372
C	-4.212464	0.060932	-5.724601
C	-4.518531	-1.255987	-6.199750
C	-4.312410	-1.210675	-7.602732
C	-3.887245	0.084462	-8.017679
Si	-5.556125	-2.569411	-5.307386
C	-6.309993	-3.726642	-6.612353
Si	-4.071534	0.756012	-9.777164
C	-5.840106	0.355540	-10.339004
C	2.932589	-3.186888	-7.817471
C	2.631591	-2.727759	-4.791199

C	0.609592	1.991981	-10.726859
C	1.209588	-1.023532	-10.788010
C	-2.859490	-0.023863	-11.004821
C	-3.871776	2.640842	-9.797656
C	-2.748116	3.208300	-4.389722
C	-1.672148	4.569631	-6.929086
C	-4.616405	-3.605032	-4.038709
C	-6.970878	-1.639111	-4.453211
C	-4.300418	-3.808796	-10.141045
C	-2.389152	-6.048026	-9.348734
C	0.102928	1.946137	-0.180605
C	-1.061146	1.914302	-0.969248
C	-2.210713	1.898015	-0.111924
C	-1.687419	1.924302	1.204112
C	-0.264691	1.942437	1.208614
Si	-3.991944	2.445750	-0.483470
C	-4.810474	1.557385	-1.944737
Si	0.662568	2.694788	2.683942
C	2.263834	1.858320	3.249738
C	-3.947612	-4.739851	-0.620387
C	-5.689016	-3.644985	1.642731
C	-1.825133	-2.433170	5.721603
C	0.922996	-1.441643	4.780226
C	2.778160	1.122072	-2.898882
C	4.001423	1.650548	-0.110315
C	-0.065213	-5.783368	0.500513
C	2.491570	-5.317124	-1.093952
C	-3.889894	4.317151	-0.804019
C	-5.091399	2.256034	1.058073
C	1.091680	4.475955	2.183982
C	-0.515139	2.753910	4.172283
H	-3.017107	-6.334433	-8.499131
H	-1.110785	-3.831774	-4.862020
H	2.563461	-1.638574	-4.846896
H	3.691379	-2.996646	-4.717556
H	2.146000	-3.051818	-3.867947
H	1.370098	-5.764480	-5.141176
H	3.009287	-5.771714	-5.812898
H	1.609483	-6.011361	-6.874841
H	3.975006	-3.459338	-7.619519
H	2.603306	-3.756402	-8.692284
H	2.912178	-2.124520	-8.075544
H	-1.360928	-6.341080	-9.114704
H	0.232500	-2.780327	-8.846871
H	-2.717999	-6.622671	-10.221757
H	1.714301	-1.870730	-10.315259
H	0.186413	-1.321127	-11.028208
H	-1.499849	-2.762099	-11.521123
H	-1.749122	-4.432202	-12.035499
H	1.727869	-0.819711	-11.731983
H	-0.365750	-4.025368	-11.014817
H	3.669358	1.003928	-10.233514
H	3.185150	1.821603	-8.736062
H	-4.458389	-2.773683	-10.454953
H	-4.995595	-4.031521	-9.326640
H	-4.574226	-4.451813	-10.985640
H	-0.438661	1.875044	-11.015714

H	1.195126	2.089738	-11.647695
H	0.705055	2.928632	-10.168881
H	-3.049906	-1.094687	-11.114742
H	-2.958835	0.435970	-11.994064
H	-2.848272	2.963141	-9.588602
H	-6.571904	0.822770	-9.672331
H	-4.140261	3.025451	-10.787725
H	-6.027993	-0.722781	-10.332546
H	-6.022701	0.722746	-11.355033
H	-4.535583	3.115532	-9.067948
H	-2.523285	4.233493	-7.528930
H	-0.820559	4.716269	-7.600728
H	-1.935915	5.543580	-6.502384
H	0.423225	3.437803	-3.664896
H	-0.059386	5.065398	-4.152501
H	1.095660	4.148059	-5.138916
H	-2.832972	4.115863	-3.783767
H	-2.646337	2.355434	-3.714260
H	-3.687237	3.089013	-4.936239
H	0.585601	0.774413	-4.703232
H	-4.260432	0.372948	-4.688306
H	-4.500481	-2.038921	-8.272194
H	-7.658419	-2.338996	-3.965593
H	-6.588861	-0.954804	-3.690400
H	-7.540934	-1.052755	-5.180898
H	-6.838354	-3.168534	-7.391645
H	-5.553014	-4.354594	-7.092990
H	-7.031288	-4.398435	-6.133842
H	-3.991003	-2.942406	-3.431455
H	-3.976605	-4.361931	-4.501412
H	-5.322776	-4.124178	-3.383117
H	-5.797086	1.183326	-1.653634
H	1.319810	-1.921325	-2.276190
H	-3.556990	4.840093	0.098718
H	-4.868300	4.720161	-1.088629
H	-3.180317	4.550502	-1.602240
H	-1.079701	1.901452	-2.051835
H	5.159837	-1.332330	-0.767653
H	1.953881	1.823999	-2.747158
H	5.639428	-0.283509	-2.113989
H	3.604298	1.669947	-3.364342
H	2.447483	0.359177	-3.608076
H	4.503709	-1.618174	-2.384413
H	4.402690	1.205409	0.805395
H	3.251049	2.395193	0.167945
H	4.824672	2.181435	-0.601641
H	-1.217471	-4.296756	-2.330197
H	0.306113	-4.288069	-3.223391
H	-0.395727	-5.826496	-2.703186
H	-5.819267	-1.124483	-0.376526
H	0.348211	-5.627230	1.500521
H	0.131313	-6.825433	0.223773
H	-4.741915	-1.602385	-1.706039
H	-3.448924	-5.406046	0.091173
H	-6.263466	-2.480415	-1.417038
H	-3.266290	-4.543716	-1.450652
H	-4.813323	-5.279476	-1.018969

H	-5.193196	-4.314456	2.353034
H	-6.030018	-2.764019	2.195837
H	-6.572744	-4.161356	1.250962
H	-1.653603	-3.876617	1.585200
H	-2.115570	0.074539	3.283622
H	-3.938421	-0.205297	1.354458
H	0.367373	-4.673945	5.169650
H	0.881228	-4.453687	3.497692
H	-0.768658	-4.996104	3.854828
H	-2.691619	-3.066597	5.507363
H	-1.388912	-2.761080	6.671611
H	-2.188458	-1.408823	5.852720
H	1.340872	-1.767046	5.739331
H	0.622400	-0.395546	4.889714
H	1.721267	-1.493835	4.034020
H	2.265274	-0.494169	1.702296
H	-2.299280	1.971161	2.095964
H	-4.615004	2.644083	1.963765
H	-5.408653	1.227679	1.252192
H	0.192792	5.023496	1.884123
H	3.007076	1.809962	2.451589
H	1.788284	4.495334	1.339511
H	1.558682	5.013795	3.016480
H	2.695187	2.450555	4.065030
H	-0.830540	1.754937	4.489919
H	-1.412870	3.342685	3.960659
H	-0.006479	3.221943	5.022107
H	-1.149741	-5.659328	0.549284
H	3.550979	0.092372	-8.723549
H	-6.001124	2.845377	0.893691
H	3.079904	-5.273465	-0.171480
H	3.015034	-4.722506	-1.848863
H	2.471763	-6.358648	-1.433742
H	-1.825621	0.100609	-10.672533
H	-4.213047	0.703372	-2.285532
H	-4.948215	2.240506	-2.787230
H	-3.179428	-4.041614	-6.504369
H	1.086828	-2.871951	1.940965
H	-1.199517	2.042399	-8.435608
H	1.737487	-0.596483	-6.668184
H	-3.590583	1.914576	-6.773637
H	1.111879	2.009656	-0.563792
H	2.095051	0.848567	3.630212

Transition state TS1

E = -3086.343909
H = -3084.457567

C	1.010777	-1.198377	9.364091
C	-0.038012	-2.106507	9.135165
C	-0.565360	-1.899731	7.819524
C	0.227814	-0.856756	7.265594
C	1.194431	-0.386346	8.199436
Th	2.154830	-3.093081	7.531846
C	4.475537	-3.770663	8.896860
C	4.879421	-3.347672	7.619903
C	4.760153	-1.921549	7.543409
C	4.239338	-1.517356	8.799686
C	4.047143	-2.640721	9.658136
Si	5.736631	-0.870961	6.295063
C	7.096758	-1.968993	5.571983
Si	3.916224	-2.609730	11.545936
C	3.876351	-0.823071	12.181160
Si	-2.384048	-2.145281	7.335641
C	-3.013185	-0.487512	6.659058
Si	1.664356	1.450535	8.221817
C	1.774365	2.140139	6.457915
O	2.163396	-2.880471	5.208348
C	2.372330	-3.423510	4.037914
O	1.849865	-2.820263	3.012503
Th	1.171892	-2.514249	0.805208
C	-1.523929	-1.979981	1.508373
C	-1.673486	-2.426143	0.154439
C	-1.344722	-3.811158	0.187036
C	-1.020712	-4.237984	1.504257
C	-1.137566	-3.065347	2.315909
Si	-1.086209	-6.054765	2.067326
C	-2.429776	-6.183812	3.397700
Si	-2.889909	-1.707695	-1.115554
C	-4.431308	-1.191234	-0.134795
C	3.014631	-0.421600	0.283994
C	2.366976	-0.087627	1.488591
C	1.020219	0.299752	1.199931
C	0.886565	0.178213	-0.213048
C	2.108060	-0.242872	-0.810333
Si	-0.053970	1.449653	2.241958
C	0.990570	2.992986	2.596768
Si	2.620694	0.334243	-2.548139
C	1.107706	0.775202	-3.598612
C	1.686100	-5.072483	-0.224915
C	2.742369	-4.765474	0.648608
C	3.579809	-3.773803	0.037436
C	2.971905	-3.486724	-1.212288
C	1.793907	-4.266625	-1.402400
Si	5.395325	-3.419491	0.470748
C	6.216824	-2.564339	-1.011429
Si	1.043501	-4.653541	-3.098278
C	2.461050	-5.303760	-4.181178
C	-1.579831	1.981981	1.249483
C	-0.593979	0.707473	3.892703

C	-3.394663	-3.095070	-2.306883
C	-2.324790	-0.216175	-2.135657
C	0.298221	-3.138882	-3.961456
C	-0.255535	-6.027594	-2.970255
C	0.533836	-6.738538	2.757531
C	-1.651921	-7.120459	0.601008
C	5.691110	-2.337148	1.992353
C	6.214044	-5.108703	0.730887
C	3.732275	-0.812013	-3.571319
C	3.611885	1.924556	-2.236014
C	0.439007	-5.300906	7.105274
C	1.675255	-5.685794	6.538187
C	2.610395	-5.958558	7.577194
C	1.901326	-5.695599	8.788323
C	0.544428	-5.346225	8.532581
Si	4.061084	-7.203399	7.514339
C	3.571745	-8.510205	6.233468
Si	-0.820655	-5.944044	9.720462
C	-1.147765	-4.935634	11.296508
C	4.687334	-0.114020	4.912901
C	6.607205	0.528544	7.239462
C	5.477272	-3.454060	12.216809
C	2.406248	-3.535175	12.220967
C	-2.761887	-3.460912	6.030506
C	-3.389019	-2.482268	8.910042
C	3.223326	1.924118	9.190282
C	0.214428	2.306336	9.104880
C	4.129499	-8.015724	9.232689
C	5.827511	-6.632513	7.128716
C	-2.434768	-6.234239	8.771424
C	-0.238708	-7.651842	10.319111
C	4.047562	-4.994749	4.066246
O	4.885707	-4.365054	4.637473
O	3.690178	-5.973159	3.482579
H	4.501315	1.720421	-1.631296
H	2.814953	-0.117167	2.475459
H	-1.348621	-0.073916	3.772612
H	-1.016043	1.489858	4.532435
H	0.261900	0.269927	4.412461
H	1.863988	2.744010	3.206982
H	0.406923	3.747229	3.135926
H	1.348943	3.441475	1.664858
H	-2.184424	2.674925	1.844660
H	-1.289329	2.502364	0.331183
H	-2.219313	1.138167	0.975130
H	3.007383	2.661379	-1.697810
H	-0.003789	0.450605	-0.766586
H	3.939777	2.375039	-3.179398
H	-2.153875	0.665584	-1.511236
H	-1.413336	-0.418272	-2.703741
H	0.556068	-0.111188	-3.922963
H	1.442604	1.303537	-4.498238
H	-3.113133	0.040971	-2.852372
H	0.416934	1.435842	-3.067039
H	-5.208831	-0.815296	-0.808941
H	-4.841347	-2.042463	0.417574
H	3.235781	-1.740254	-3.863492

H	4.662013	-1.066462	-3.055327
H	4.005071	-0.284397	-4.492730
H	-2.552485	-3.487367	-2.884058
H	-4.135108	-2.717409	-3.020417
H	-3.850515	-3.928391	-1.762980
H	1.079581	-2.431318	-4.252248
H	-0.220432	-3.448748	-4.875563
H	-1.173068	-5.716815	-2.463213
H	2.894382	-6.211853	-3.750259
H	-0.533601	-6.357168	-3.977533
H	3.262837	-4.563881	-4.270420
H	2.106585	-5.541506	-5.190288
H	0.148731	-6.896814	-2.441939
H	-0.928523	-7.150100	-0.218815
H	-2.605532	-6.766098	0.196659
H	-1.797732	-8.150750	0.944260
H	-2.171367	-5.610966	4.291372
H	-2.563191	-7.229548	3.696266
H	-3.389463	-5.809533	3.027773
H	0.335460	-7.597759	3.407007
H	1.071329	-5.989452	3.343346
H	1.208958	-7.071817	1.965070
H	-0.939304	-3.012935	3.379301
H	2.873605	-5.152882	1.648858
H	3.377461	-2.807895	-1.950188
H	7.274559	-4.994195	0.979950
H	5.731537	-5.663325	1.541150
H	6.139441	-5.714878	-0.177715
H	6.072354	-3.126037	-1.939329
H	5.836051	-1.548470	-1.156839
H	7.295007	-2.485343	-0.833625
H	5.076138	-2.643678	2.839946
H	5.495896	-1.279978	1.790625
H	6.739319	-2.427829	2.296306
H	4.308631	-9.320862	6.219749
H	0.059843	-0.420795	6.288038
H	4.433191	-7.311119	10.014958
H	4.865240	-8.827432	9.225481
H	3.161212	-8.439009	9.515966
H	1.871283	-5.775226	5.477501
H	-2.830791	0.318908	7.376338
H	-2.611725	-4.478172	6.398755
H	-4.093100	-0.543821	6.481522
H	-3.806498	-3.372782	5.711820
H	-2.142355	-3.325159	5.139437
H	-2.533389	-0.212923	5.716416
H	-3.307124	-1.637086	9.600883
H	-3.080010	-3.384102	9.442776
H	-4.447042	-2.597805	8.648752
H	2.173304	1.404089	5.756200
H	0.788669	2.447625	6.097728
H	2.425075	3.020484	6.432433
H	7.728149	-2.369858	6.371735
H	3.250421	1.478937	10.189102
H	3.220007	3.012563	9.319997
H	6.685554	-2.808396	5.008084
H	4.106964	0.733106	5.288111

H	7.734389	-1.381331	4.902237
H	3.995259	-0.853334	4.501762
H	5.329532	0.250240	4.104364
H	5.911344	1.238184	7.694794
H	7.243313	0.121907	8.031975
H	7.246928	1.092707	6.551294
H	4.047271	-0.493678	9.089014
H	4.518970	-4.789616	9.254996
H	5.244782	-3.980826	6.825330
H	3.920886	-0.823625	13.275747
H	2.964172	-0.291208	11.892954
H	4.734261	-0.252398	11.812313
H	6.375734	-2.916186	11.898339
H	5.467990	-3.485731	13.311785
H	5.560353	-4.483011	11.852116
H	2.400922	-3.504340	13.316110
H	2.427479	-4.586048	11.917260
H	1.468409	-3.096763	11.868904
H	-0.422545	-2.808651	9.861536
H	2.296426	-5.881407	9.781990
H	6.215226	-5.922759	7.864367
H	5.934719	-6.188312	6.136593
H	-2.246210	-6.838052	7.877946
H	-1.561047	-3.937840	11.128084
H	-2.936236	-5.315745	8.461444
H	-3.129658	-6.792314	9.408762
H	-1.872626	-5.488107	11.905940
H	0.678070	-7.584933	10.913369
H	-0.040122	-8.315270	9.471559
H	-1.008140	-8.119478	10.943782
H	4.146883	1.657993	8.671225
H	-4.208733	-0.401714	0.590120
H	6.463646	-7.525178	7.165599
H	0.111464	1.946777	10.133760
H	-0.728715	2.107630	8.586391
H	0.363008	3.391564	9.138849
H	-0.421493	-2.614730	-3.326422
H	3.525984	-8.078768	5.229106
H	2.593444	-8.942417	6.465378
H	4.052689	-0.713727	0.191179
H	1.553945	-1.093835	10.294724
H	-1.402567	-4.470935	-0.669897
H	-1.732061	-0.980334	1.863717
H	0.919697	-5.813677	-0.046168
H	-0.461099	-5.081652	6.544341
H	-0.235106	-4.836960	11.891324

Complex C3

E = -3086.440849
H = -3084.553846

Th	0.875258	-2.267530	0.394649
C	2.551824	-0.047906	-0.093195
H	2.683258	2.900692	-2.114576
C	0.367828	0.425856	-0.480226
C	0.558697	0.526853	0.925089
C	1.940169	0.217636	1.144625
C	1.583318	0.077304	-1.139642
C	1.865596	-4.853011	-0.102739
C	2.794654	-4.292334	0.792761
C	3.547842	-3.280469	0.120017
C	3.033879	-3.251579	-1.207058
C	1.999340	-4.218329	-1.381459
C	-1.329572	-4.018044	0.929356
C	-1.429142	-3.757578	-0.465180
C	-1.785312	-2.397458	-0.706676
C	-1.899308	-1.806185	0.593793
C	-1.623850	-2.774077	1.574621
Si	-0.592077	1.465850	2.106175
Si	-2.798467	-1.843456	-2.208016
Si	1.696334	-5.073808	-3.044469
Si	-1.487214	-5.720303	1.747466
Si	5.266400	-2.645568	0.642326
Si	2.027542	0.612636	-2.903089
O	1.284871	-2.410088	2.621145
C	1.148576	-2.683899	3.884371
O	0.082400	-2.840739	4.464517
Th	2.132434	-3.237384	8.175381
C	0.207798	-5.264390	8.388381
C	0.828353	-5.496713	7.148926
C	2.143701	-6.022199	7.380700
C	2.281036	-6.083614	8.789967
C	1.110152	-5.608321	9.446865
C	4.758654	-3.984131	8.782407
C	4.962599	-2.674825	8.247998
C	4.427389	-1.781807	9.215732
C	3.948744	-2.494783	10.358481
C	4.160928	-3.871325	10.055991
C	0.118091	-1.871349	9.596781
C	-0.533147	-2.081166	8.335786
C	0.173715	-1.279782	7.405910
C	1.218332	-0.549467	8.051797
C	1.151300	-0.927421	9.423876
Si	6.306572	-2.264035	6.972015
C	5.959293	-0.700745	5.971126
Si	3.863840	-1.830146	12.138650
C	4.094956	-3.303120	13.312066
C	-2.002821	2.241799	1.101143
C	-1.345875	0.430463	3.503620
C	0.433138	2.880866	2.841903
C	3.780290	0.069509	-3.376293
C	1.996598	2.511662	-2.872998
C	0.816502	0.031748	-4.234561

C	-0.119051	-6.112694	2.989182
C	-1.534107	-7.074791	0.419719
C	-3.148094	-5.720103	2.662676
C	-2.145783	-2.546589	-3.838448
C	-2.970382	0.039447	-2.347244
C	-4.540788	-2.544911	-1.921210
C	5.343116	-1.152512	1.802843
C	6.123153	-4.102182	1.499031
C	6.212334	-2.236528	-0.953805
C	1.727977	-3.853293	-4.491227
C	0.099149	-6.088543	-3.125567
C	3.150944	-6.277443	-3.251967
Si	-2.318040	-2.661654	8.061807
C	-2.542016	-3.794797	6.561946
Si	1.926479	1.059668	7.358329
C	3.343128	1.760587	8.409127
Si	3.187957	-7.068729	6.194936
C	2.230247	-8.691987	5.955363
Si	0.652518	-6.049270	11.230744
C	-0.781740	-7.289453	11.145975
C	7.880912	-1.989449	8.002377
C	6.573819	-3.711717	5.790384
C	2.311851	-0.889470	12.701382
C	5.337589	-0.651671	12.335515
C	-2.993139	-3.484680	9.635850
C	-3.328420	-1.081964	7.764649
C	0.510653	2.324769	7.420667
C	2.472799	0.845599	5.562581
C	4.865607	-7.495080	6.972498
C	3.456204	-6.268747	4.508029
C	2.129342	-6.904376	12.058898
C	0.104091	-4.586257	12.304162
H	2.440074	0.192542	2.105528
H	-2.182618	-0.175656	3.144206
H	-1.729440	1.094242	4.286460
H	-0.621167	-0.250971	3.956062
H	1.294234	2.506762	3.401962
H	-0.174370	3.486093	3.523520
H	0.809119	3.534364	2.048193
H	-2.601286	2.888162	1.752597
H	-1.614423	2.858064	0.284411
H	-2.680267	1.497624	0.671462
H	0.994727	2.884760	-2.637275
H	-0.565658	0.644505	-0.984402
H	2.291535	2.924392	-3.844236
H	-3.320021	0.483890	-1.410372
H	-2.039519	0.536111	-2.633036
H	0.884015	-1.046622	-4.395065
H	1.053739	0.524490	-5.183916
H	-3.716155	0.272650	-3.115456
H	-0.218438	0.280505	-3.984293
H	-5.213165	-2.275392	-2.743354
H	-4.516913	-3.636717	-1.846241
H	3.875197	-1.014244	-3.488436
H	4.515240	0.404453	-2.637847
H	4.051920	0.523204	-4.335912
H	-1.162554	-2.143651	-4.091720

H	-2.832021	-2.289408	-4.652763
H	-2.073970	-3.637666	-3.803794
H	2.590060	-3.181278	-4.440533
H	1.793710	-4.402236	-5.436929
H	-0.793975	-5.460092	-3.173016
H	3.176585	-7.005590	-2.435031
H	0.115008	-6.705004	-4.031357
H	4.104803	-5.740633	-3.247647
H	3.073961	-6.828014	-4.196174
H	-0.002826	-6.764303	-2.271932
H	-0.565436	-7.219365	-0.067882
H	-2.276106	-6.855523	-0.354145
H	-1.809205	-8.028067	0.884284
H	-3.151726	-4.974797	3.463924
H	-3.343157	-6.699429	3.113466
H	-3.972627	-5.487655	1.981464
H	-0.338214	-7.051202	3.509814
H	-0.050572	-5.313293	3.731790
H	0.852537	-6.218590	2.498750
H	-1.607295	-2.611167	2.646032
H	2.928162	-4.572920	1.830405
H	3.426915	-2.625837	-2.000034
H	7.167161	-3.856501	1.722367
H	5.626173	-4.340176	2.443713
H	6.109935	-4.996565	0.868443
H	6.273890	-3.110188	-1.610579
H	5.748344	-1.423080	-1.520629
H	7.234674	-1.925535	-0.711883
H	4.690894	-1.319244	2.663056
H	5.075324	-0.206950	1.324216
H	6.370791	-1.054297	2.170940
H	4.013675	-5.332517	4.580848
H	-0.063224	-1.222452	6.350095
H	2.083995	-9.203165	6.912278
H	2.770678	-9.370418	5.285845
H	1.242195	-8.505615	5.522880
H	0.379272	-5.309146	6.180691
H	-3.211303	-0.379328	8.595602
H	-2.229199	-4.824261	6.757477
H	-4.394224	-1.313574	7.659983
H	-3.599639	-3.817349	6.276380
H	-1.960433	-3.421987	5.714136
H	-3.000816	-0.577802	6.850102
H	-2.893432	-2.817589	10.498551
H	-2.497487	-4.429399	9.878535
H	-4.059863	-3.699000	9.507465
H	3.271068	0.106910	5.469884
H	1.630020	0.518834	4.945696
H	2.839338	1.795023	5.158124
H	6.766732	-4.650008	6.319149
H	3.107652	1.726542	9.477896
H	3.498719	2.812001	8.143139
H	5.694418	-3.828698	5.151928
H	5.642881	0.138846	6.596542
H	7.436414	-3.513747	5.145014
H	5.186782	-0.901028	5.224957
H	6.867172	-0.394033	5.440205

H	7.759512	-1.144938	8.688424
H	8.117251	-2.874800	8.601199
H	8.738874	-1.778626	7.354185
H	4.458477	-0.702180	9.137250
H	3.933832	-4.697061	10.718376
H	5.063086	-4.910237	8.311952
H	5.405872	-0.283266	13.364940
H	5.244030	0.216719	11.675214
H	6.276164	-1.157857	12.090142
H	5.013596	-3.850901	13.080459
H	4.166238	-2.949459	14.346369
H	3.258190	-4.006626	13.263399
H	2.400318	-0.714294	13.779939
H	1.385429	-1.445255	12.533214
H	2.213037	0.090450	12.224804
H	-0.179386	-2.310264	10.542175
H	3.153300	-6.473409	9.301535
H	4.767716	-7.877303	7.993460
H	5.543963	-6.637592	6.987310
H	-0.499833	-8.169249	10.559098
H	-0.807849	-4.122325	11.916293
H	-1.666145	-6.846980	10.676633
H	-1.067442	-7.625842	12.148771
H	-0.110748	-4.933226	13.320996
H	2.990395	-6.240993	12.184300
H	2.454949	-7.777256	11.484248
H	1.839164	-7.254037	13.055729
H	4.290704	1.240086	8.248736
H	-4.969270	-2.158032	-0.991159
H	5.346476	-8.277863	6.375392
H	0.182448	2.491936	8.451734
H	-0.354093	1.972712	6.849967
H	0.825179	3.287917	7.003495
H	0.818083	-3.248652	-4.522887
H	2.495631	-6.056012	4.031323
H	4.010325	-6.951282	3.854400
H	3.597111	-0.283761	-0.238011
H	1.788174	-0.546940	10.208438
H	-1.311345	-4.508537	-1.237126
H	-2.187504	-0.781211	0.788871
H	1.188360	-5.667975	0.122300
H	-0.805955	-4.918681	8.522926
H	0.879787	-3.818130	12.370487
C	2.494817	-2.821090	4.674541
O	3.558585	-2.789287	4.078550
O	2.365050	-2.982203	5.961289

Transition state TS2

E = -3274.908058
H = -3273.003901

Th	0.957034	-2.648448	0.813112
C	1.536118	-5.217843	-0.116387
C	1.618587	-4.409474	-1.294571
C	2.794352	-3.618739	-1.118952
C	3.432544	-3.913815	0.116074
C	2.612579	-4.914810	0.736499
C	-1.520265	-4.003991	0.329923
C	-1.861364	-2.624879	0.222951
C	-1.700711	-2.100511	1.547364
C	-1.299017	-3.134270	2.410035
C	-1.171909	-4.349406	1.666219
O	3.237272	-5.202394	3.853489
C	2.166105	-3.148229	4.171453
O	1.552633	-2.779334	3.124512
C	3.441094	-3.982974	4.049252
O	4.506382	-3.346516	4.179295
O	1.749224	-2.853671	5.350553
Th	1.983788	-3.124525	7.676277
C	0.395708	-5.392273	7.315802
C	1.531775	-5.625304	6.514249
C	2.662687	-5.857985	7.351781
C	2.166836	-5.749345	8.686851
H	2.761212	-5.930882	9.575014
C	0.766625	-5.494600	8.696133
C	-0.588342	-2.440458	8.910925
C	0.395352	-1.876640	9.785258
C	0.147628	-0.475891	9.892735
C	-0.965647	-0.223731	9.039483
C	-1.444870	-1.416707	8.441711
O	1.253454	-0.701754	7.099790
C	0.809891	0.370782	7.345795
O	0.603544	1.521145	7.304471
Si	-0.407864	-6.002180	10.106628
C	-0.134131	-5.070992	11.729253
Si	4.225167	-6.855417	6.853191
C	3.637772	-8.123552	5.579458
C	3.846349	-1.438831	8.989089
C	4.252911	-1.428268	7.628635
C	4.691305	-2.760475	7.339274
C	4.589535	-3.530739	8.515463
C	4.049518	-2.731976	9.568352
Si	0.885036	-4.881584	-2.976745
C	2.302269	-5.655724	-3.972779
Si	5.300270	-3.668092	0.462221
C	6.128833	-3.602173	-1.248854
Si	-3.120642	-1.961195	-1.034835
C	-3.483187	-3.292968	-2.332988
Si	-1.209446	-6.118718	2.371135
C	-2.250173	-6.030353	3.953154
Si	4.725800	0.083120	6.578582
C	6.607912	-0.012762	6.374273
Si	4.354409	-3.108825	11.406690

C	5.888623	-2.097937	11.880204
Si	-3.109679	-1.573241	7.580984
C	-3.618398	0.095218	6.835869
Si	0.647183	0.807107	11.188517
C	2.492996	1.257940	11.264262
C	4.770916	-7.766488	8.431777
C	5.723973	-5.936363	6.161303
C	-2.208949	-5.817070	9.550235
C	-0.057169	-7.843194	10.403984
C	0.219861	-3.401981	-3.955617
C	-0.471976	-6.191458	-2.783027
C	5.839827	-2.133511	1.429485
C	5.878863	-5.212690	1.382214
C	-4.709814	-1.655224	-0.042230
C	-2.666396	-0.343752	-1.904845
C	0.452168	-6.926156	2.777522
C	-2.116390	-7.202481	1.103870
C	3.933483	0.049381	4.867195
C	4.299749	1.682427	7.501458
C	4.751642	-4.945719	11.650049
C	2.944508	-2.627440	12.571160
C	-3.071601	-2.901958	6.230493
C	-4.433207	-2.054993	8.858687
C	-0.285155	2.419095	10.822288
C	0.102049	0.190442	12.901231
C	2.228751	-0.263826	1.513765
C	2.767077	-0.558380	0.249225
C	1.768784	-0.348750	-0.753521
C	0.605298	0.067281	-0.039145
C	0.864017	0.139443	1.358473
Si	-0.128197	1.159056	2.607382
C	0.974151	2.620639	3.102246
Si	2.199140	0.250123	-2.504302
C	0.676074	0.446141	-3.609483
C	3.512490	-0.790267	-3.390976
C	2.944108	1.977868	-2.242041
C	-1.682048	1.857081	1.769756
C	-0.604585	0.198730	4.165975
H	2.845303	1.657958	10.308418
H	-2.327234	-2.662355	5.465911
H	-2.615985	-3.528232	-2.955397
H	-1.573314	-7.273011	0.155622
H	0.780219	-5.968914	0.069404
H	3.850651	1.927510	-1.630871
H	2.770063	-0.318606	2.450612
H	-1.475006	-0.443289	4.005759
H	-0.851914	0.904380	4.965343
H	0.218218	-0.438376	4.501763
H	1.896036	2.280491	3.583486
H	0.452583	3.281236	3.803311
H	1.253727	3.208670	2.222391
H	-2.213037	2.510713	2.470388
H	-1.416552	2.458154	0.894017
H	-2.385388	1.082813	1.448519
H	2.234679	2.635830	-1.730336
H	-0.328849	0.355256	-0.506669
H	3.205469	2.439635	-3.200724

H	-2.529723	0.474484	-1.190787
H	-1.756983	-0.436484	-2.502677
H	0.198742	-0.508122	-3.844715
H	0.976809	0.908777	-4.556110
H	-3.481197	-0.055825	-2.578638
H	-0.068185	1.101723	-3.147274
H	-5.521947	-1.328250	-0.700897
H	-5.032486	-2.567136	0.469689
H	3.140224	-1.759808	-3.732223
H	4.385322	-0.962718	-2.753479
H	3.859984	-0.242263	-4.273974
H	-4.281944	-2.946666	-2.997964
H	-3.825585	-4.218499	-1.859332
H	1.031307	-2.732457	-4.255424
H	-0.278496	-3.743610	-4.869448
H	-1.396199	-5.796680	-2.352565
H	2.705020	-6.533194	-3.457196
H	-0.720229	-6.600455	-3.768724
H	3.123070	-4.944332	-4.108592
H	1.959475	-5.970710	-4.964581
H	-0.129722	-7.024058	-2.160338
H	-3.116980	-6.813386	0.890790
H	-2.225220	-8.220230	1.494308
H	-1.784130	-5.379276	4.698566
H	-2.358151	-7.025642	4.397569
H	-3.249642	-5.635529	3.747286
H	0.281635	-7.744793	3.485948
H	1.173754	-6.236817	3.225610
H	0.923049	-7.360286	1.890261
H	-1.105313	-3.022014	3.469019
H	2.814787	-5.371968	1.699540
H	3.184287	-2.933354	-1.860248
H	6.958961	-5.165090	1.558839
H	5.377652	-5.292669	2.351128
H	5.666369	-6.116854	0.802946
H	5.878890	-4.484712	-1.845847
H	5.830626	-2.715564	-1.818218
H	7.218043	-3.567236	-1.135056
H	5.350190	-2.104461	2.405764
H	5.673459	-1.191167	0.899983
H	6.918708	-2.218013	1.606471
H	3.351317	-7.606988	4.659614
H	-1.444455	0.743924	8.936596
H	5.113628	-7.084908	9.217578
H	5.605730	-8.436304	8.197408
H	3.959211	-8.374500	8.843645
H	1.567186	-5.645489	5.432485
H	-3.667920	0.872633	7.605284
H	-2.825691	-3.882460	6.651032
H	-4.611980	0.019183	6.380884
H	-4.045173	-2.992132	5.737216
H	-2.918904	0.427551	6.064397
H	-4.482445	-1.312644	9.661741
H	-4.217662	-3.025457	9.316633
H	-5.423116	-2.116328	8.392533
H	-0.056893	2.804210	9.823715
H	-1.368444	2.282705	10.895897

H	0.002774	3.185208	11.550497
H	7.112061	-0.028718	7.345637
H	3.136335	0.418653	11.542975
H	2.636263	2.039628	12.019074
H	6.890517	-0.919251	5.830308
H	2.848476	0.173797	4.922758
H	6.980767	0.849632	5.810745
H	4.154971	-0.915979	4.398965
H	4.344578	0.848728	4.241102
H	3.224412	1.852488	7.600958
H	4.744370	1.690117	8.501783
H	4.712753	2.535434	6.951584
H	3.471946	-0.575773	9.525556
H	4.896930	-4.562430	8.615765
H	5.054993	-3.103708	6.375692
H	6.169680	-2.280409	12.923228
H	5.706397	-1.025120	11.761955
H	6.740444	-2.363008	11.246170
H	5.554819	-5.279225	10.985968
H	5.090636	-5.106446	12.679414
H	3.882051	-5.589516	11.489017
H	3.264118	-2.788098	13.606957
H	2.042250	-3.222287	12.407488
H	2.679216	-1.572291	12.469685
H	-0.769933	-3.500653	8.778272
H	6.245473	-5.332901	6.909459
H	5.459354	-5.301487	5.311666
H	-2.392821	-6.339639	8.606308
H	-0.345714	-4.003320	11.625583
H	-2.502574	-4.770615	9.431879
H	-2.867014	-6.259610	10.306189
H	-0.800392	-5.474223	12.499853
H	0.969359	-8.001309	10.749706
H	-0.192360	-8.419741	9.483618
H	-0.734651	-8.249214	11.163252
H	-4.562152	-0.881364	0.717861
H	6.432560	-6.694315	5.805774
H	0.598342	-0.743388	13.181369
H	-0.977197	0.006950	12.908440
H	0.324591	0.935900	13.672899
H	-0.500483	-2.819935	-3.374327
H	2.781495	-8.697001	5.947779
H	4.444367	-8.825971	5.342029
H	3.786817	-0.867806	0.064452
H	1.048717	-2.446633	10.445986
H	-1.584052	-4.712704	-0.486149
H	-1.915088	-1.083383	1.846089
H	-0.608688	-5.218587	6.944569
H	0.892435	-5.195182	12.088045

Complex C4

E = -3274.978459
H = -3273.073186

Th	1.039666	-2.690094	0.750136
C	2.482991	-0.380795	1.379337
C	2.886367	-0.661107	0.062645
C	1.809585	-0.380085	-0.835740
C	0.739193	0.068424	-0.004559
C	1.129684	0.088337	1.363650
C	-1.530243	-2.066208	1.696038
C	-1.808951	-2.515104	0.363599
C	-1.527177	-3.912057	0.379198
C	-1.105986	-4.340402	1.670191
C	-1.121460	-3.160513	2.478277
C	1.459702	-5.259142	-0.267454
C	1.542017	-4.429621	-1.431353
C	2.756359	-3.694477	-1.276219
C	3.411692	-4.038704	-0.063210
C	2.569863	-5.019083	0.560211
O	1.786161	-2.913525	2.980315
C	2.354308	-3.306952	4.046559
O	1.945410	-2.958407	5.208237
C	3.579672	-4.227182	3.965471
O	3.343850	-5.396281	3.591614
O	4.649341	-3.684061	4.315795
Th	2.285791	-3.289392	7.549354
C	0.682758	-5.519758	7.193922
C	1.837183	-5.808238	6.430902
C	2.933553	-6.048041	7.309858
C	2.403300	-5.883937	8.626433
C	1.010973	-5.585869	8.583916
C	4.871025	-3.503369	8.508552
C	4.243948	-2.739222	9.540232
C	3.955668	-1.473812	8.947641
C	4.398235	-1.436366	7.595882
C	4.961877	-2.724435	7.333794
C	-2.612485	-1.540331	10.227884
C	-1.758474	-0.880849	11.206370
C	-0.717116	-0.258017	10.597405
C	-2.137116	-1.357407	8.970167
C	-0.820028	-0.586531	9.112532
C	0.319892	-1.476910	8.598511
H	-1.194062	-2.363227	5.952628
O	0.863452	-1.230977	7.474901
Si	2.111404	0.232678	-2.607494
C	3.314202	-0.839497	-3.605788
Si	0.300135	1.122095	2.718411
C	-1.278301	1.912545	2.018504
Si	5.279047	-3.819308	0.284133
C	5.803652	-2.415792	1.439367
Si	0.716583	-4.830742	-3.088387
C	0.011795	-3.318646	-3.985957
Si	-1.188886	-6.139113	2.292343
C	0.450537	-7.008332	2.655966
Si	-3.129690	-1.749548	-0.765836

C	-4.655499	-1.510351	0.339185
Si	4.514098	-7.032536	6.862977
C	5.016959	-7.955038	8.447732
Si	-0.237286	-5.844490	9.989418
C	-1.970790	-5.395704	9.377127
Si	4.628021	0.105738	6.518298
C	4.175817	-0.258986	4.725462
Si	4.394586	-3.094578	11.396742
C	4.428104	-4.954566	11.758843
Si	0.256085	1.122168	11.451159
C	2.024608	1.321037	10.819738
Si	-3.125203	-1.698482	7.396005
C	-2.176802	-2.787321	6.178648
O	0.702531	-2.501368	9.255764
C	-2.713786	-0.070431	-1.532438
C	-3.586133	-2.975684	-2.137114
C	-2.136460	-7.142565	0.988684
C	-2.223513	-6.093201	3.881673
C	2.928169	1.934695	-2.393842
C	0.511983	0.492512	-3.584859
C	-0.116031	0.170725	4.298572
C	1.512246	2.520302	3.138279
C	-0.655630	-6.118497	-2.858205
C	2.063987	-5.595776	-4.183562
C	5.875716	-5.454046	1.021893
C	6.097963	-3.537600	-1.408474
C	6.008453	-6.071494	6.219775
C	3.986629	-8.273124	5.536269
C	-0.183027	-7.701153	10.385878
C	0.146260	-4.868772	11.561235
C	3.022215	-2.266002	12.402933
C	6.065269	-2.367420	11.929906
C	3.566842	1.523961	7.185680
C	6.462383	0.576564	6.630309
C	-4.743011	-2.553488	7.883817
C	-3.512024	-0.026050	6.589019
C	0.267875	0.804464	13.318156
C	-0.671521	2.741462	11.091861
H	-2.759336	-3.178671	-2.822571
H	2.969101	-6.047968	9.536147
H	-1.603222	-7.193321	0.033796
H	0.676867	-5.979870	-0.073065
H	3.883005	1.850912	-1.865547
H	3.105256	-0.488841	2.259364
H	-1.031814	-0.416799	4.187780
H	-0.269575	0.874980	5.123103
H	0.681607	-0.515401	4.593054
H	2.450764	2.125158	3.537930
H	1.082914	3.193617	3.888397
H	1.749174	3.109577	2.246813
H	-1.721501	2.575888	2.769362
H	-1.056391	2.516566	1.132829
H	-2.039950	1.175948	1.744360
H	2.285587	2.605796	-1.815238
H	-0.219680	0.412874	-0.373262
H	3.117685	2.400433	-3.367443
H	-2.516749	0.686546	-0.766898

H	-1.849810	-0.122518	-2.198329
H	-0.036746	-0.435984	-3.759448
H	0.753698	0.923950	-4.562709
H	-3.569572	0.277083	-2.122188
H	-0.150234	1.197583	-3.073188
H	-5.504799	-1.139947	-0.245605
H	-4.950341	-2.454187	0.808268
H	2.891702	-1.804676	-3.896642
H	4.242497	-1.023770	-3.055832
H	3.583218	-0.307005	-4.524946
H	-4.413432	-2.568628	-2.728638
H	-3.920139	-3.928539	-1.714370
H	0.805612	-2.641731	-4.313470
H	-0.541178	-3.633156	-4.877922
H	-1.534752	-5.719261	-2.344927
H	2.476729	-6.495764	-3.717083
H	-0.985116	-6.474095	-3.840778
H	2.889960	-4.894561	-4.340138
H	1.664715	-5.872851	-5.165476
H	-0.294223	-6.987121	-2.298924
H	-3.126788	-6.716376	0.799624
H	-2.272988	-8.170810	1.341116
H	-1.730175	-5.500612	4.657558
H	-2.368066	-7.105377	4.274625
H	-3.208383	-5.652029	3.699490
H	0.263509	-7.845326	3.338305
H	1.198080	-6.356091	3.116521
H	0.898505	-7.424907	1.748661
H	-0.852413	-3.109653	3.525457
H	2.777217	-5.495554	1.512822
H	3.156026	-3.011721	-2.015206
H	6.956839	-5.422306	1.195889
H	5.381450	-5.640659	1.980685
H	5.660941	-6.293011	0.352335
H	5.840523	-4.335523	-2.111904
H	5.804549	-2.583189	-1.858986
H	7.187730	-3.523497	-1.296832
H	5.419363	-2.586275	2.448838
H	5.505798	-1.420546	1.099273
H	6.898527	-2.425442	1.501308
H	3.726318	-7.731431	4.621745
H	-0.811576	0.312318	8.485504
H	5.327255	-7.272214	9.246150
H	5.863992	-8.617842	8.239167
H	4.195161	-8.568752	8.829777
H	1.894813	-5.864310	5.350493
H	-4.077451	0.616995	7.270868
H	-2.041299	-3.789285	6.595451
H	-4.109642	-0.165997	5.681580
H	-2.731404	-2.885867	5.240081
H	-2.595370	0.499878	6.305488
H	-5.343813	-1.930601	8.553942
H	-4.557204	-3.511835	8.378618
H	-5.342800	-2.752599	6.989201
H	-0.698127	2.955297	10.018204
H	-1.704345	2.692595	11.450375
H	-0.178360	3.585421	11.587041

H	6.758479	0.766591	7.666840
H	2.646608	0.469694	11.104815
H	2.472290	2.223343	11.250252
H	7.093646	-0.229401	6.243056
H	3.087561	-0.280732	4.616276
H	6.669462	1.479591	6.045508
H	4.567611	-1.234735	4.420181
H	4.578552	0.511990	4.059766
H	2.509239	1.242553	7.193473
H	3.861641	1.804878	8.201411
H	3.677094	2.410063	6.551369
H	3.492797	-0.640882	9.462805
H	5.243706	-4.513773	8.618364
H	5.360979	-3.056625	6.380606
H	6.233006	-2.514298	13.002658
H	6.107354	-1.293244	11.723550
H	6.889557	-2.842641	11.388781
H	5.155078	-5.478557	11.130313
H	4.722019	-5.115973	12.801846
H	3.450582	-5.422415	11.616148
H	3.024839	-2.655007	13.427050
H	2.034223	-2.444433	11.971125
H	3.182610	-1.185606	12.465172
H	-3.535721	-2.052853	10.481037
H	6.497267	-5.465632	6.988221
H	5.737428	-5.423703	5.380215
H	-2.234467	-5.944324	8.467246
H	0.067064	-3.796519	11.367973
H	-2.042662	-4.322525	9.179020
H	-2.713343	-5.646736	10.142567
H	-0.567687	-5.137243	12.347942
H	0.810906	-8.001220	10.733092
H	-0.419815	-8.297938	9.499380
H	-0.904802	-7.954857	11.170234
H	2.053081	1.424431	9.731392
H	-4.457270	-0.788151	1.137692
H	6.746461	-6.800608	5.864203
H	0.762299	-0.141686	13.557491
H	-0.744942	0.775963	13.732114
H	0.808917	1.607402	13.830386
H	-0.671432	-2.752858	-3.346640
H	3.122587	-8.863879	5.855799
H	4.807514	-8.960927	5.305278
H	3.870709	-1.002744	-0.229769
H	-1.981576	-0.841187	12.268529
H	-1.681119	-4.574604	-0.463624
H	-1.665131	-1.055906	2.058730
H	-0.306409	-5.338215	6.788235
H	1.149483	-5.081187	11.942153

Complex C5

E = -3275.027998
H = -3273.121999

Th	-3.670303	-2.132270	0.246238
C	-0.940271	-2.805254	0.829428
O	-1.334081	-2.521271	-0.363029
O	-1.828635	-2.949412	1.733393
C	0.499505	-2.810603	1.138761
C	1.546723	-3.107606	0.282011
C	2.761853	-2.626992	0.913453
C	2.473026	-2.028553	2.101297
C	1.041675	-2.222503	2.398200
Si	1.134647	-3.483864	3.911768
C	2.060028	-2.591895	5.308834
C	2.072599	-5.042146	3.395922
Si	1.680475	-4.102489	-1.335956
C	2.318993	-2.967700	-2.711923
C	2.997606	-5.436882	-1.014753
C	0.081879	-4.965533	-1.839500
Si	-5.126291	-2.038756	4.235305
C	-7.015200	-2.080102	4.120943
C	-4.455342	-1.037532	2.774171
C	-3.151234	-0.507873	2.588732
C	-3.131761	0.423349	1.507539
C	-3.361890	-1.362959	-2.523828
C	-5.270847	-0.408343	1.782246
C	-4.478458	0.472220	1.026938
Si	-1.867844	-0.738648	-3.510849
C	-2.495767	0.678703	-4.609514
C	-0.423529	-0.096447	-2.473099
Si	-1.889575	1.817969	1.224739
C	-0.087606	1.236675	1.171639
C	-2.059542	3.022550	2.684500
H	-2.380199	2.101382	-1.235208
C	-2.349553	2.755223	-0.359423
C	-4.436583	-0.560735	-2.045952
C	-5.577717	-1.360217	-1.754120
C	-5.185035	-2.698523	-2.050187
C	-3.851037	-2.704480	-2.506689
Si	-7.374949	-0.786809	-1.595775
C	-8.387459	-1.969789	-0.522703
C	-8.093491	-0.800901	-3.355497
C	-7.503179	0.988796	-0.939556
C	-1.270318	-2.145571	-4.632379
C	-4.471872	-3.814524	4.329856
H	-3.379828	-3.836885	4.315675
H	1.591732	-2.190474	-2.958467
H	1.761834	-5.375538	2.401841
H	3.151411	-4.869082	3.368096
H	1.608930	-1.619406	5.532973
H	3.178208	-1.550809	2.771556
H	2.008252	-3.194424	6.222555
H	-0.525127	-4.572121	5.397758
H	-1.111992	-2.989477	4.859814
H	0.494621	-1.366649	2.806234

H	-1.192532	-4.365352	3.756819
H	-2.289770	-0.771205	3.191377
H	-0.110455	-0.849805	-1.745831
H	0.226106	0.865284	2.152480
H	0.083462	0.445629	0.437570
H	0.568300	2.077707	0.920433
H	-1.817227	2.527706	3.630599
H	-1.387281	3.880317	2.569766
H	-3.083799	3.400854	2.760756
H	-1.621864	3.549102	-0.558821
H	-3.332145	3.227440	-0.256565
H	-1.694058	1.046770	-5.259370
H	-2.850494	1.524225	-4.010939
H	-3.323616	0.347124	-5.244063
H	-4.409880	0.521399	-1.982054
H	-4.837544	1.115263	0.234899
H	-7.962647	-2.083716	0.477464
H	-9.417796	-1.609988	-0.427219
H	-8.425163	-2.968338	-0.969198
H	-8.032600	-1.802753	-3.792523
H	-9.145820	-0.495442	-3.353426
H	-8.534259	1.341151	-1.055338
H	-7.242475	1.071328	0.118838
H	-5.828469	-3.565978	-1.963076
H	-3.291309	-3.580783	-2.811553
H	-6.329977	-0.587233	1.642416
H	-2.078830	-2.498165	-5.280560
H	-0.453098	-1.796402	-5.272858
H	-3.509067	-1.085374	5.906602
H	-4.983109	-1.644054	6.711208
H	-4.979017	-0.106241	5.828318
C	-4.599886	-1.133039	5.821066
H	0.252002	-5.561649	-2.742672
H	2.520490	-3.547624	-3.619149
H	2.689129	-6.115661	-0.213234
H	3.152959	-6.036161	-1.918867
H	3.962365	-5.003019	-0.733809
Si	-8.915807	-7.605914	1.857092
C	-7.886804	-8.379940	0.443393
C	-7.260792	-8.703263	-1.761870
C	-7.849085	-9.772043	0.149824
C	-7.473559	-10.006882	-1.206810
H	-4.802201	-10.559015	-3.091085
C	-5.715353	-9.476129	2.734056
H	-6.750317	-9.355276	3.021658
C	-3.906851	-8.601170	-2.543723
C	-2.892088	-8.309433	-1.577644
Si	-4.354898	-7.493125	-4.016283
H	-7.498512	-7.556641	3.969175
H	-8.766900	-6.332062	3.957083
H	-7.307783	-6.074759	2.995031
C	-2.715835	-6.847867	-4.716803
H	-2.890723	-6.212724	-5.591949
H	-2.175339	-6.253800	-3.973299
H	-2.066820	-7.675589	-5.019625
C	-5.432960	-6.010102	-3.559548
H	-4.984988	-5.476362	-2.718340

H	-5.495662	-5.309723	-4.398866
H	-6.451197	-6.295090	-3.281533
H	-9.423800	-5.431659	0.754134
H	-10.761970	-5.934955	1.801475
C	-10.023775	-6.287672	1.072688
H	-10.563473	-6.684324	0.207124
C	-7.505355	-7.730948	-0.774115
C	-10.019212	-9.002932	2.527807
H	-10.691804	-8.601506	3.293938
H	-10.635804	-9.439223	1.735554
H	-9.443793	-9.811259	2.990750
Si	-8.124814	-11.487343	-2.196746
C	-7.239329	-11.771396	-3.846508
H	-7.184207	-10.860253	-4.448793
H	-7.797518	-12.513641	-4.428022
C	-9.934863	-11.057280	-2.577436
H	-10.504127	-10.913269	-1.653771
H	-10.415478	-11.856444	-3.152617
H	-10.004626	-10.131646	-3.157417
H	-6.225504	-12.156711	-3.710771
C	-8.097820	-13.095533	-1.198482
H	-7.089635	-13.512053	-1.129618
H	-8.486154	-12.956970	-0.185034
H	-8.728951	-13.842380	-1.692421
H	-7.906721	-13.272516	3.754767
H	-8.224965	-12.209242	2.383420
C	-7.605240	-12.334400	3.275851
H	-7.837501	-11.522720	3.972104
C	-3.975444	-13.624423	-1.974996
H	-4.738741	-13.680946	-1.195848
Si	-5.754012	-12.400867	2.879334
H	-3.531697	-14.620235	-2.082154
H	-4.465289	-13.382089	-2.922779
C	-5.430278	-13.817558	1.670656
H	-5.143654	-13.719019	4.926208
H	-6.000446	-13.689055	0.748025
H	-5.741485	-14.764445	2.125525
H	-4.371415	-13.908160	1.413917
C	-4.816741	-12.769142	4.488834
C	-1.697861	-12.896085	0.005690
H	-4.984151	-11.978804	5.227261
H	-2.355878	-13.037882	0.866738
H	-1.192268	-13.849576	-0.183171
H	-0.927103	-12.169305	0.280966
Si	-2.626482	-12.365368	-1.559017
C	-1.372148	-12.355114	-2.985348
H	-0.930127	-13.347520	-3.127928
H	-0.560853	-11.647437	-2.787454
H	-1.850011	-12.058321	-3.924357
C	-3.745692	-10.390620	2.079424
H	-2.987198	-11.110918	1.797308
C	-5.118893	-10.705124	2.311419
C	-3.280931	-10.589254	-1.463115
C	-3.483525	-9.020553	2.347149
C	-2.519430	-9.498380	-0.929403
C	-4.741439	-8.463273	2.748936
H	-1.750181	-9.586351	-0.172435

H	-4.914987	-7.434798	3.042434
Si	-1.768677	-8.297817	2.713974
C	-1.875815	-7.569785	4.460375
H	-0.896980	-7.204337	4.787846
H	-2.577089	-6.730916	4.502983
H	-0.925361	-7.411134	0.531355
C	-1.162548	-6.977047	1.506880
H	-0.245342	-6.527281	1.900760
H	-1.890576	-6.177274	1.351847
H	-2.213684	-8.328162	5.173658
H	0.468092	-9.348924	3.027214
C	-0.517107	-9.724303	2.729708
H	-0.403126	-10.192677	1.746900
H	-0.809282	-10.501623	3.442477
H	-2.487319	-7.325999	-1.370645
C	-4.126905	-10.003712	-2.451182
C	-8.015628	-6.832319	3.334646
H	-7.425941	-6.657817	-0.893460
H	-4.630165	-9.409779	-5.616634
C	-5.220520	-8.528163	-5.348496
H	-6.213333	-8.866007	-5.036745
H	-7.438278	-1.070796	4.096484
H	-7.334500	-2.612945	3.220251
H	-7.437930	-2.599691	4.987626
H	-4.812561	-4.290502	5.256031
H	-4.839652	-4.405907	3.487865
H	3.116701	-2.432501	5.075525
H	3.754235	-2.722946	0.484865
H	0.426069	0.152617	-3.118782
H	-0.697375	0.810748	-1.927864
H	3.250742	-2.475780	-2.414503
H	-0.713635	-4.242078	-2.024274
H	-0.257958	-5.636956	-1.045712
H	1.873327	-5.851386	4.106415
H	-8.137499	-10.551296	0.845779
H	-5.350884	-7.925052	-6.253630
H	-3.738894	-12.833513	4.308543
O	-4.694513	-6.671112	0.214772
C	-4.585058	-5.402870	0.272065
O	-3.572561	-4.742604	-0.047970
C	-5.803122	-4.578276	0.769422
O	-5.489721	-3.347708	1.015439
O	-6.903273	-5.092473	0.879227
Th	-5.115123	-8.951645	0.024278
C	-0.592102	-3.896698	4.537905
H	-6.998145	-8.499859	-2.792362
H	-6.861120	1.672233	-1.505299
H	-0.900311	-2.998601	-4.056705
H	-7.542836	-0.117958	-4.010427

Complex C6

E = -3275.026937
H = -3273.119834

C	-3.034506	-0.562612	1.932527
C	-3.944704	-1.544233	2.409335
C	-5.115302	-1.399969	1.606280
C	-4.914863	-0.355081	0.681539
C	-3.610366	0.194673	0.869615
Th	-3.255110	-2.474154	-0.196701
C	-3.203244	-3.033952	-2.975414
C	-2.910195	-1.636694	-2.941165
C	-4.120302	-1.005686	-2.524112
C	-5.150053	-1.964585	-2.325956
C	-4.545714	-3.228891	-2.600222
Si	-3.759826	-2.541578	4.008790
C	-3.846463	-4.414600	3.749527
Si	-3.075056	1.955137	0.433940
C	-1.207758	2.165693	0.659752
Si	-1.524605	-0.817240	-3.940891
C	-0.545585	-2.171259	-4.832600
Si	-7.011770	-1.607178	-2.281928
C	-7.300071	0.260470	-2.462632
O	-1.735327	-3.379959	1.450621
C	-0.654419	-2.943866	0.913311
C	0.619955	-3.326017	1.537072
C	2.036890	-2.920750	1.258412
C	2.783100	-3.648888	2.311536
C	1.938029	-4.384865	3.095214
C	0.611092	-4.183227	2.619639
O	-0.768861	-2.197789	-0.127482
C	-7.905338	-2.246736	-0.738203
C	-7.765742	-2.454755	-3.803431
C	-2.369247	0.276839	-5.243323
C	-0.357916	0.250545	-2.910151
C	-3.965127	3.091902	1.668519
C	-3.580802	2.506570	-1.308788
C	-2.112562	-2.087072	4.823910
C	-5.188284	-2.023920	5.148889
O	-2.659192	-4.852498	-0.663004
C	-3.485749	-5.773313	-0.418535
C	-4.858927	-5.337301	0.077072
O	-5.011755	-4.113990	0.349472
O	-3.340218	-7.009770	-0.542069
Th	-5.314058	-8.696703	-0.285363
C	-7.984469	-9.390913	0.674021
C	-8.002750	-7.979075	0.585200
C	-7.923423	-7.688670	-0.817609
C	-7.887515	-8.892730	-1.534511
C	-7.903518	-9.991120	-0.618167
Si	-8.700603	-6.816749	1.912488
C	-7.526376	-5.567945	2.711933
Si	-8.589466	-11.704953	-1.031388
C	-10.323588	-11.794555	-0.260172
C	-10.106888	-5.849991	1.080363
C	-9.429007	-7.885932	3.302675

C	-8.808625	-11.907183	-2.906137
C	-7.573605	-13.146040	-0.340513
C	-3.971922	-8.209155	2.139541
C	-5.185945	-8.794617	2.543853
C	-5.169943	-10.187774	2.218177
C	-3.914076	-10.400623	1.571713
C	-3.146049	-9.203683	1.530772
Si	-1.254460	-9.173106	1.388761
C	-0.488132	-7.921250	0.201100
Si	-5.941615	-11.525739	3.330588
C	-5.296849	-11.103854	5.067737
C	-7.831558	-11.624500	3.469032
C	-5.295246	-13.246219	2.872647
C	-0.625335	-8.772960	3.136969
C	-0.652031	-10.913085	0.926110
C	-4.192096	-10.758064	-2.059767
C	-5.287170	-10.210585	-2.781479
C	-5.025357	-8.862021	-3.169851
C	-3.720426	-8.579402	-2.668421
C	-3.233603	-9.694662	-1.970886
Si	-3.701178	-12.592219	-2.151812
C	-2.045016	-12.631779	-3.081064
Si	-5.846259	-7.909593	-4.587984
C	-6.820034	-9.142309	-5.651234
C	-4.971328	-13.517301	-3.215310
C	-3.452575	-13.530367	-0.524808
C	-4.447156	-7.153088	-5.622041
C	-7.009534	-6.495578	-4.086161
O	-5.725269	-6.242715	0.158209
H	-0.040329	-2.839314	-4.129772
H	-9.392546	-12.813703	-3.101401
H	-7.876775	-8.981506	-2.612452
H	-6.720491	-6.061084	3.262557
H	-3.702321	-7.171087	2.295584
H	-5.171299	-0.945155	5.333821
H	-0.286103	-4.625070	3.034720
H	-5.963367	-13.560786	-2.759115
H	-4.257904	0.064952	-2.436788
H	-7.632026	-9.618202	-5.092108
H	-5.674862	-10.133098	5.403832
H	-0.811132	-8.062234	-0.832029
H	0.602109	-8.025184	0.241316
H	-0.747160	-6.897167	0.481412
H	-0.966367	-7.782806	3.456193
H	0.469785	-8.778022	3.168997
H	-0.992324	-9.502103	3.866085
H	0.443072	-10.936818	0.929685
H	-1.002533	-11.662286	1.643108
H	-0.984219	-11.214612	-0.071755
H	-4.203518	-11.055699	5.077411
H	-3.559881	-11.367275	1.234050
H	-5.612433	-11.859882	5.795359
H	-2.611178	-13.127827	0.046826
H	-4.339222	-13.515712	0.112132
H	-5.690606	-13.600873	1.917762
H	-5.606937	-13.959381	3.643954
H	-3.221590	-14.577532	-0.751232

H	-4.202768	-13.269844	2.823292
H	-1.733008	-13.665696	-3.266376
H	-2.127872	-12.121776	-4.045768
H	-8.317758	-11.951739	2.546494
H	-8.282303	-10.677007	3.777582
H	-8.069133	-12.362720	4.244005
H	-4.634972	-14.548167	-3.372232
H	-5.068532	-13.045094	-4.198024
H	-7.653538	-13.189724	0.748977
H	-7.939298	-14.099652	-0.736691
H	-7.867675	-12.004672	-3.454200
H	-10.969917	-11.009603	-0.665657
H	-10.285336	-11.664131	0.826018
H	-10.793374	-12.762616	-0.467050
H	-9.364734	-11.063224	-3.326725
H	-6.164443	-9.933442	-6.028281
H	-7.266563	-8.633373	-6.512288
H	-3.894396	-6.396729	-5.055272
H	-4.850302	-6.665255	-6.516104
H	-3.734499	-7.919012	-5.943027
H	-7.231044	-5.885709	-4.968977
H	-6.546001	-5.841771	-3.341208
H	-7.959568	-6.853011	-3.680438
H	-3.178970	-7.653521	-2.802908
H	-7.932090	-6.696055	-1.250137
H	-8.079310	-9.942490	1.598575
H	-10.627605	-5.218479	1.808460
H	-9.721912	-5.197774	0.289946
H	-10.838461	-6.528371	0.630584
H	-10.109280	-8.648916	2.912485
H	-8.644178	-8.388482	3.877837
H	-9.991282	-7.257942	4.002313
H	-7.075172	-4.913622	1.963456
H	-8.088190	-4.952568	3.424169
H	-7.668709	-3.543039	-3.746730
H	-6.948369	0.838223	-1.602361
H	-8.373091	0.455041	-2.567563
H	-6.802741	0.647637	-3.357603
H	-5.052259	-4.186299	-2.563561
H	-6.157922	-2.271726	4.704853
H	-3.007092	-4.744803	3.132040
H	-5.122357	-2.536012	6.115252
H	-4.780040	-4.695797	3.254857
H	-3.802216	-4.935564	4.712344
H	-1.271282	-2.351298	4.176891
H	-2.055741	-1.016220	5.043908
H	-1.995793	-2.628575	5.768913
H	-2.049765	-0.384982	2.349956
H	-5.648589	-0.001365	-0.031106
H	-6.012756	-2.000396	1.692414
H	-3.688738	4.140273	1.510112
H	-3.708822	2.823737	2.698280
H	-5.051765	3.008315	1.564720
H	-4.650012	2.353002	-1.486735
H	-3.382708	3.578447	-1.420698
H	-3.024683	1.984774	-2.092175
H	-0.920651	3.205841	0.470802

H	-0.640860	1.529353	-0.024354
H	-0.903637	1.919525	1.681972
H	-7.724248	-1.606530	0.129778
H	-7.578820	-3.258662	-0.482202
H	-1.203230	-2.772800	-5.467985
H	0.104865	-0.356997	-2.130074
H	0.220139	-1.722140	-5.474458
H	0.433998	0.678988	-3.533823
H	-2.946645	1.081406	-4.776642
H	-3.054363	-0.311233	-5.862040
H	-1.625629	0.737840	-5.902925
H	-1.249023	-12.141056	-2.512164
H	-8.986207	-2.267650	-0.916113
H	-6.515854	-13.052719	-0.597237
H	-7.270231	-2.121747	-4.720826
H	-8.832344	-2.218130	-3.886659
H	-5.982962	-8.280138	3.067143
H	2.219736	-5.016854	3.929577
H	-6.168869	-10.769083	-3.066430
H	-2.253771	-9.763892	-1.516679
H	-2.508230	-3.817630	-3.252938
H	-0.888824	1.076813	-2.429026
H	3.861095	-3.599502	2.418662
Si	2.738729	-3.660522	-0.410847
Si	2.418101	-1.032483	1.587116
C	1.779339	-3.092950	-1.930005
C	4.558593	-3.167038	-0.637625
C	2.639429	-5.545678	-0.289853
C	1.291518	-0.464799	2.995380
C	4.217200	-0.801057	2.148337
C	2.130725	0.033807	0.058986
H	2.807823	-5.988954	-1.277245
H	3.388168	-5.948511	0.397718
H	1.653124	-5.863234	0.057802
H	4.947780	-3.694440	-1.516246
H	4.684539	-2.095560	-0.819608
H	5.183803	-3.448776	0.214845
H	2.161892	-3.631664	-2.804588
H	0.713809	-3.304301	-1.822145
H	1.893201	-2.022129	-2.113393
H	2.234993	1.091926	0.322373
H	2.847398	-0.184948	-0.738224
H	1.122747	-0.126647	-0.327121
H	1.594540	0.528972	3.342709
H	0.250215	-0.407385	2.668918
H	1.344918	-1.152337	3.844708
H	4.420476	0.273066	2.228415
H	4.393972	-1.245580	3.132194
H	4.942108	-1.220430	1.445705

Complex C7

E = -3275.042595
H = -3273.136022

Th	-3.216747	-2.427940	0.083663
O	-1.301331	-3.207108	1.465794
C	-0.471764	-2.461371	0.854927
O	-0.904435	-1.693669	-0.082300
C	0.951890	-2.457877	1.205032
C	1.539372	-3.262836	2.149695
C	2.983973	-2.976349	2.218136
C	3.161235	-1.903999	1.201702
C	1.966881	-1.612822	0.616791
Si	3.940922	-4.577536	1.655436
C	3.585700	-4.850567	-0.182047
C	5.809164	-4.372383	1.902574
C	2.120396	-0.947867	4.407006
C	5.118597	-1.390051	3.915035
Si	3.427121	-2.243969	3.966050
C	3.447119	-3.588906	5.301014
C	-3.834506	-1.855473	2.783920
C	-5.014522	-1.557550	2.036495
C	-4.788143	-0.398758	1.263652
C	-3.462846	0.076594	1.506296
H	0.475545	-2.721031	-2.157525
C	-2.699110	-1.638765	-2.603203
C	-3.760604	-0.793045	-2.161472
C	-3.263771	-2.942745	-2.700366
C	-4.625524	-2.882475	-2.342128
C	-4.968100	-1.539059	-2.011980
Si	-6.748101	-0.892577	-1.973495
C	-7.799266	-1.657583	-0.593456
C	-7.521599	-1.373016	-3.639824
C	-6.772615	1.000680	-1.848346
Si	-1.041592	-1.150863	-3.379216
Si	-2.763508	1.815677	1.250595
C	-3.739315	-8.411076	1.854658
C	-4.204303	-10.315648	-2.667480
C	-5.390164	-9.740237	-3.202847
C	-4.896059	-9.108695	2.248436
C	-3.907250	-8.044752	-2.974606
C	-5.234867	-8.336155	-3.405511
C	-2.912861	-9.271172	1.067263
C	-3.623421	-10.500023	0.989821
C	-4.842849	-10.442389	1.732293
C	-7.849101	-8.163937	0.650840
C	-7.788345	-9.575723	0.557709
C	-7.933850	-8.803244	-1.567420
C	-7.823711	-10.007063	-0.801129
Si	-8.419990	-7.189623	2.174117
Si	-8.500096	-11.677345	-1.378760
C	-8.873590	-11.634680	-3.239280
H	-9.430962	-12.537374	-3.513447
C	-7.400729	-13.169679	-0.990281
Si	-5.461918	-11.959209	2.698184
C	-7.327079	-12.160077	2.987194

C	-4.961380	-12.927248	-4.127086
C	-4.800879	-13.563757	1.938775
C	-4.670958	-11.770245	4.416278
C	-3.234851	-13.226064	-1.587618
Si	-3.643715	-12.092101	-3.047693
C	-2.066698	-11.915252	-4.090864
Si	-1.034972	-9.132519	0.817588
C	-0.312487	-9.005269	2.571345
C	-0.411210	-10.748447	0.038823
C	-0.362583	-7.680964	-0.188784
Si	-6.240594	-7.250493	-4.592284
C	-4.991806	-6.301949	-5.659788
C	-7.403579	-5.976956	-3.797648
H	-8.032339	-8.757034	-2.643821
C	-7.273721	-8.389390	-5.703137
C	-9.949473	-6.204267	1.633168
C	-8.935173	-8.431309	3.515535
H	-6.305870	-6.480046	3.344338
Si	-3.593075	-3.096544	4.192540
C	-2.019948	-2.611651	5.130162
C	-5.098597	-2.975105	5.339269
C	-3.417813	-4.884383	3.591812
C	-1.379516	-0.878600	-5.230514
C	-3.609597	2.925038	2.540447
C	-3.105332	2.558783	-0.458438
C	-0.898066	1.798397	1.577448
Th	-5.253685	-8.629352	-0.521527
C	-7.931139	-7.700534	-0.704818
C	-3.297330	-9.217995	-2.502919
C	-2.901569	-0.839033	2.438373
O	-2.615311	-4.759167	-0.423680
C	-3.459788	-5.697390	-0.353322
O	-3.298625	-6.906870	-0.614917
C	-10.153544	-11.926101	-0.476982
C	-7.192675	-5.976062	2.949234
C	-0.363688	0.457462	-2.651101
C	0.213635	-2.557065	-3.205269
C	3.348009	-6.094741	2.622594
C	-4.863521	-5.308823	0.094746
O	-5.720074	-6.231148	0.068707
O	-5.045009	-4.111354	0.437987
H	-3.502601	-7.391764	2.137035
H	-5.218390	-1.956699	5.721963
H	1.782809	-0.882062	-0.161052
H	1.019931	-4.000554	2.749772
H	-5.914955	-13.062117	-3.609757
H	-3.678612	0.277863	-2.018318
H	-8.014342	-8.960466	-5.134235
H	-5.038018	-10.873865	4.926263
H	-0.699027	-7.701432	-1.227602
H	0.732766	-7.733320	-0.180323
H	-0.663820	-6.716842	0.227431
H	-0.646074	-8.086552	3.065029
H	0.782742	-8.994086	2.544263
H	-0.629912	-9.849847	3.190932
H	0.681975	-10.728023	-0.028349
H	-0.689677	-11.618423	0.642111

H	-0.798996	-10.897490	-0.973648
H	-3.582724	-11.683917	4.337270
H	-3.250124	-11.392103	0.501899
H	-4.899441	-12.636144	5.047781
H	-2.373168	-12.860043	-1.021909
H	-4.073945	-13.343770	-0.898718
H	-5.259977	-13.795672	0.974850
H	-5.019814	-14.396043	2.617084
H	-2.976442	-14.219468	-1.971531
H	-3.716189	-13.527002	1.801739
H	-1.714041	-12.895988	-4.428821
H	-2.252241	-11.296391	-4.974232
H	-7.887356	-12.373640	2.073528
H	-7.775860	-11.286868	3.469321
H	-7.467695	-13.010531	3.664592
H	-4.610925	-13.918255	-4.436032
H	-5.145332	-12.340906	-5.032837
H	-7.355225	-13.351043	0.086710
H	-7.807453	-14.071912	-1.460201
H	-7.977233	-11.604887	-3.864970
H	-10.846275	-11.109813	-0.705222
H	-10.014257	-11.947578	0.608577
H	-10.627235	-12.868145	-0.774954
H	-9.498905	-10.773473	-3.495505
H	-6.635248	-9.102632	-6.233686
H	-7.815660	-7.799561	-6.450240
H	-4.438136	-5.562746	-5.072053
H	-5.507126	-5.763252	-6.462412
H	-4.266684	-6.982647	-6.115975
H	-7.743865	-5.276142	-4.568499
H	-6.902260	-5.395368	-3.018356
H	-8.290165	-6.439742	-3.356193
H	-3.431440	-7.074181	-3.008649
H	-8.001363	-6.663612	-1.005255
H	-7.770354	-10.239310	1.410270
H	-10.385417	-5.664352	2.480848
H	-9.691848	-5.467062	0.865834
H	-10.716130	-6.866061	1.218348
H	-9.658232	-9.158435	3.133514
H	-8.076973	-8.981564	3.915496
H	-9.400542	-7.902219	4.354313
H	-6.868097	-5.228197	2.222231
H	-7.680109	-5.460917	3.785051
H	-7.555348	-2.460396	-3.760954
H	-6.406976	1.375650	-0.888028
H	-7.799547	1.362218	-1.970512
H	-6.166414	1.450924	-2.640937
H	-5.315686	-3.718120	-2.346207
H	-6.017464	-3.245335	4.809186
H	-2.547034	-4.983119	2.936827
H	-4.995392	-3.651374	6.194864
H	-4.314605	-5.193218	3.047745
H	-3.289965	-5.563210	4.442342
H	-1.149227	-2.691013	4.471849
H	-2.077388	-1.585594	5.506732
H	-1.859990	-3.277493	5.984971
H	-1.900992	-0.762539	2.847876

H	-5.518392	0.077146	0.622509
H	-5.933002	-2.132042	2.054038
H	-3.221094	3.948568	2.495466
H	-3.444923	2.542404	3.552619
H	-4.690841	2.966541	2.373061
H	-4.162414	2.491462	-0.732974
H	-2.834159	3.620434	-0.452162
H	-2.513131	2.071062	-1.236683
H	-0.472976	2.792680	1.403274
H	-0.384871	1.088091	0.923246
H	-0.679478	1.523209	2.614212
H	-7.554737	-1.244696	0.389024
H	-7.656254	-2.741244	-0.544269
H	-0.177932	-3.494967	-3.612542
H	-0.234075	0.362491	-1.570514
H	1.129533	-2.316904	-3.756358
H	0.606593	0.698918	-3.099031
H	-2.111965	-0.079637	-5.384704
H	-1.775655	-1.787758	-5.694213
H	-0.460447	-0.602219	-5.759454
H	-1.256790	-11.447639	-3.521707
H	-8.861074	-1.464024	-0.781681
H	-6.379856	-13.028632	-1.352815
H	-6.940470	-0.962600	-4.471633
H	-8.546510	-0.993923	-3.718964
H	-5.676688	-8.711443	2.886574
H	4.113860	-1.439616	0.972126
H	-6.266243	-10.306426	-3.490746
H	-2.280773	-9.284800	-2.136855
H	-2.729583	-3.836562	-3.000197
H	-1.034202	1.298812	-2.853533
H	2.454612	-0.360787	5.269382
H	1.956835	-0.260898	3.571524
H	1.161919	-1.407835	4.662318
H	3.564053	-3.117623	6.283375
H	2.514952	-4.161897	5.318990
H	4.278456	-4.287937	5.169336
H	5.352342	-1.005191	4.914199
H	5.928484	-2.061479	3.619740
H	5.110874	-0.538803	3.227723
H	6.326716	-5.226695	1.452109
H	6.188075	-3.465173	1.422027
H	6.083022	-4.340543	2.961180
H	3.923095	-6.969939	2.299452
H	3.480974	-5.990704	3.702251
H	2.292103	-6.302794	2.425880
H	3.971358	-5.827113	-0.494887
H	2.510270	-4.832487	-0.380826
H	4.056890	-4.083778	-0.802612

6. References

[1] R. R. Langeslay, M. E. Fieser, J. W. Ziller, F. Furche, W. J. Evans, *Chem. Sci.* **2015**, *6*, 517–521.

[2] C. A. P. Goodwin, F. Tuna, E. J. L. McInnes, S. T. Liddle, J. McMaster, I. J. Vitorica-Yrezabal, D. P. Mills, *Chem. Eur. J.* **2014**, *20*, 14579–14583.

[3] P. B. Hitchcock, M. F. Lappert, L. Maron, A. V. Protchenko, *Angew. Chem. Int. Ed.* **2008**, *47*, 1488–1491; *Angew. Chem.* **2008**, *120*, 1510–1513.

[4] *CrysAlis Pro*, Agilent Technologies: Yarnton, England, **2010**.

[5] a) G. M. Sheldrick, *Acta Cryst. Sect. A* **2008**, *64*, 112–122; b) G. M. Sheldrick, *Acta Cryst., Sect. C.* **2015**, *71*, 3–8.

[6] M. C. Burla, R. Caliandro, M. Camalli, B. Carrozzini, G. L. Cascarano, L. De Caro, C. Giacovazzo, G. Polidori, D. Siliqi, R. Spagna, *J. Appl. Cryst.*, **2007**, *40*, 609–613.

[7] Olex2: O. V. Dolomanov, L. J. Bourhis, R. J. Gildea, J. A. K. Howard, H. Puschmann, *J. Appl. Cryst.*, **2009**, *42*, 339–341.

[8] L. J. Farrugia, *J. Appl. Cryst.*, **2012**, *45*, 849–854.

[9] *POV-Ray*, Persistence of Vision Raytracer Pty. Ltd.: Williamstown, Australia, **2004**.

[10] A. L. Spek, *Platon*; University of Utrecht, Utrecht, The Netherlands, **2000**.

[11] R. Ahlrichs, M. Bär, M. Häser, H. Horn, C. Kölmel, *Chem. Phys. Lett.* **1989**, *162*, 165–169.

[12] A. D. Becke, *J. Chem. Phys.* **1993**, *98*, 5648.

[13] P. Stephens, F. Devlin, C. Chabalowski, M. Frisch, *J. Phys. Chem.* **1994**, *98*, 11623.

[14] R. Ditchfield, W. J. Hehre, J. A. Pople, *J. Chem. Phys.* **1971**, *54*, 724.

[15] W. Küchle, M. Dolg, H. Stoll, H. Preuss, *J. Chem. Phys.* **1994**, *100*, 7535.

[16] X. Cao, M. Dolg, *J. Mol. Struct. THEOCHEM* **2004**, *673*, 203–209.

[17] D. A. Pantazis, F. Neese, *J. Chem. Theory Comput.* **2011**, *7*, 677–684.

[18] T. A. Keith, *AIMAll (Version 14.11.23), TK Gristmill Software, Overl. Park KS, USA* **2014**.

[19] T. Lu, F. Chen, *J. Comput. Chem.* **2012**, *33*, 580–592.

[20] Gaussian 09, Revision **D.01**, M. J. Frisch, G. W. Trucks, H. B. Schlegel, G. E. Scuseria, M. A. Robb, J. R. Cheeseman, G. Scalmani, V. Barone, B. Mennucci, G. A. Petersson, H. Nakatsuji, M.

Caricato, X. Li, H. P. Hratchian, A. F. Izmaylov, J. Bloino, G. Zheng, J. L. Sonnenberg, M. Hada, M. Ehara, K. Toyota, R. Fukuda, J. Hasegawa, M. Ishida, T. Nakajima, Y. Honda, O. Kitao, H. Nakai, T. Vreven, J. A. Montgomery, Jr., J. E. Peralta, F. Ogliaro, M. Bearpark, J. J. Heyd, E. Brothers, K. N. Kudin, V. N. Staroverov, R. Kobayashi, J. Normand, K. Raghavachari, A. Rendell, J. C. Burant, S. S. Iyengar, J. Tomasi, M. Cossi, N. Rega, M. J. Millam, M. Klene, J. E. Knox, J. B. Cross, V. Bakken, C. Adamo, J. Jaramillo, R. Gomperts, R. E. Stratmann, O. Yazyev, A. J. Austin, R. Cammi, C. Pomelli, J. W. Ochterski, R. L. Martin, K. Morokuma, V. G. Zakrzewski, G. A. Voth, P. Salvador, J. J. Dannenberg, S. Dapprich, A. D. Daniels, Ö. Farkas, J. B. Foresman, J. V. Ortiz, J. Cioslowski, D. J. Fox, Gaussian, Inc., Wallingford CT, **2009**.

[21] A. Moritz, X. Cao, M. Dolg, *Theor. Chem. Acc.* **2007**, *118*, 845–854.

[22] W. J. Hehre, R. Ditchfield, J. A. Pople, *J. Chem. Phys.* **1972**, *56*, 2257.

[23] K. Burke, J. P. Perdew, W. Yang, in *Electronic Density Functional Theory: Recent Progress and New Directions* (Eds.: J. F. Dobson, G. Vignale, M. P. Das), Plenum, New York, **1998**.

[24] A. E. Reed, L. A. Curtiss, F. Weinhold, *Chem. Rev.* **1988**, *88*, 899–926.
